# Messenger RNA profile analysis deciphers new Esrrb responsive genes in prostate cancer cells

**DOI:** 10.1186/s12867-015-0049-1

**Published:** 2015-12-01

**Authors:** Yuan Lu, Jilong Li, Jianlin Cheng, Dennis B. Lubahn

**Affiliations:** Department of Biochemistry, University of Missouri, Columbia, MO 65211 USA; MU Center for Botanical Interaction Studies, University of Missouri, Columbia, MO 65211 USA; Computer Science Department, University of Missouri, Columbia, MO 65211 USA; Informatics Institute, University of Missouri, Columbia, MO 65211 USA; Xiphophorus Genetic Stock Center, Texas State University, San Marcos, TX 78666 USA

**Keywords:** Estrogen related receptor, RNA-Seq, Prostate cancer, Gene expression profile, DY131, Transcription regulation, Nuclear receptor

## Abstract

**Background:**

Orphan nuclear receptor estrogen related receptor β (Esrrb or ERRβ) is well known in stem cells and early embryonic development. However, little is known about its function in cancer.

**Method:**

We investigated the mRNA profile alterations induced by Esrrb expression and its synthetic ligand DY131 in human prostate cancer DU145 cells via RNA-Seq analysis.

**Results:**

We distinguished 67 mRNAs differentially expressed by Esrrb alone. Although DY131 alone did not change any gene, treatment of DY131 in the presence of Esrrb altered 1161 mRNAs. These observations indicated Esrrb had both ligand-independent and ligand-dependent activity. When Esrrb was expressed, DY131 treatment further regulated 15 Esrrb-altered mRNAs. DY131 acted as an antagonist for 11 of 15 mRNAs (*wdr52*, *f13a1*, *pxdn*, *spns2*, *loc100506599*, *tagln*, *loc441454*, *tkel1*, *sema3f*, *zcwpw2*, *sdc2*) and as an agonist for 4 of the 15 mRNAs (*rarres3*, *oasl*, *padi2*, *ddx60*). Gene ontology analyses showed altered genes are related to transcription and translation regulation, cell proliferation and apoptosis regulation, and cellular metabolism.

**Conclusion:**

Our results characterized mRNA profiles in DU145 prostate cancer cells driven by Esrrb expression and Esrrb ligand DY131, and provided multiple markers to characterize Esrrb’s function in Esrrb research.

**Electronic supplementary material:**

The online version of this article (doi:10.1186/s12867-015-0049-1) contains supplementary material, which is available to authorized users.

## Background

Esrrb encodes nuclear receptor estrogen related receptor β (Esrrb), which belongs to the nuclear receptor family. Esrrb acts as a transcription factor by binding to a specific DNA sequence estrogen related receptor response element (ERRE), which is also known as steroid factor response element (SFRE), or half site estrogen response element [1, 2].

Esrrb, first cloned in 1988, was not intensively studied until recent years. Knocking out of Esrrb was embryonic lethal due to placental malformation [3]. Though early studies showed a very limited range of tissues with positive Esrrb expression, recent studies reported that short form Esrrb alternative splicing isoform had a broad range of expression [4]. Esrrb was found to be a core-reprogramming factor to reprogram Pluripotent Stem Cells (iPSCs) [3–6]. *c*-*myc* and *klf4* of the OSKM (*oct4*, *sox2*, *klf4*, *c*-*myc*) core-reprogramming factors can be replaced by Esrrb [5, 6]. Esrrb was also recently reported to drive *sox2* transcription and induce iPSC in a single cell system [7].

Tumorigenesis and tumor progression are related to Esrrb. Esrrb was shown to be down-regulated in prostate cancer epithelium compared to normal prostate tissue [8–10]. Its re-expression in DU145 and LNCaP cells was shown to stimulate tumor suppressor *cdkn1a* (p21) concentration. Also, Esrrb can inhibit Estrogen Receptor transcriptional activity in uterine endometrial cancer cells and Nrf2-Keap signaling pathway in breast cancer cells [11, 12].

There are a handful of transcriptome-wide expression survey data from Esrrb knockdown in both human iPSCs and mouse embryonic stem cells [13–16]. Known Esrrb controlled genes include *klf4*, *c*-*myc*, *cdkn1a* and *cyp19a1*, but Esrrb target genes in cancer cells are still not known.

This manuscript focuses on the discovery of Esrrb ligand-independent and Esrrb ligand-dependent target genes. We performed RNA-Seq analysis to characterize Esrrb regulated mRNAs in a prostate cancer cell line and we found the treatment of DY131 expanded Esrrb’s transcriptional regulation activity to many more genes.

## Results

### Establishment of the Esrrb stably transfected DU145 cells

**Fig. 1:**
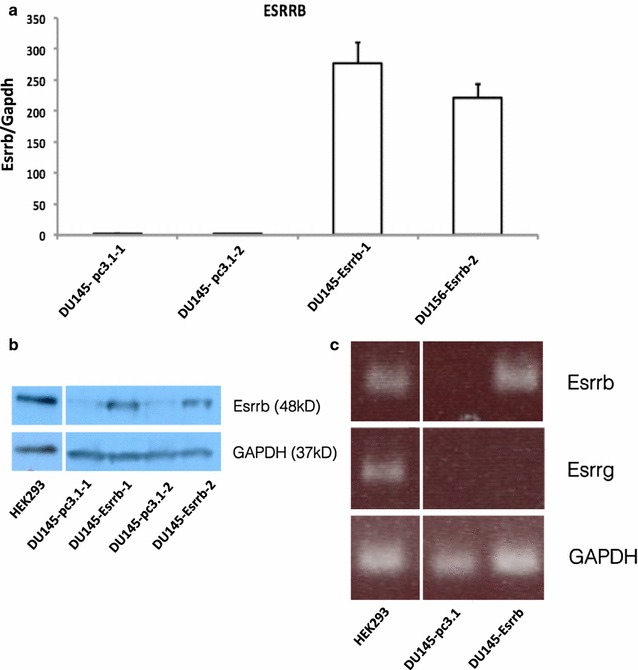
Characterization of Esrrb-expressing cancer cell line. Esrrb status of two independent replicates of stable transfected control DU145-pc3.1 and DU145-Esrrb cells are tested by **a** quantitative PCR **b** Western blot and **c** reverse transcriptase PCR. **a** Relative mRNA concentrations of Esrrb were measured by qPCR, Esrrb transcripts concentration were determined by standard curve method and Esrrb concentration were first normalized to the concentration of house keeping gene GAPDH, then normalized to Esrrb/GAPDH ratio of DU145-pc3.1 cells. **b** Total protein was extracted form HEK293, DU145-Esrrb and control DU145-pc3.1 cells. Protein concentration of Esrrb was determined by western blot using GAPDH as internal control. **c** RT-PCR was performed on total RNA extracted from HEK293, DU145-esrrb and control DU145-pc3.1 cells. Esrrb was expressed in DU145-Esrrb cells, while Esrrg is not expressed in either DU145-pc3.1 and DU145-Esrrb cells

Esrrb expression vector or control pcDNA3.1 (Zeo+) vector were transfected into DU145 cells. After 3 weeks of Zeocine selection, we characterized the Esrrb status by reverse transcriptase (RT)-PCR, qPCR and western blot analysis (Fig. 1a–c). Our results showed that Esrrb was successfully expressed in DU145-Esrrb cells. Although RNA-Seq showed that DU145-pc3.1 cells had a very small amount of Esrrb expressed (count per million read <1), the Esrrb concentration is below the detection limit of RT-PCR and western blot. Compared to HEK293 cells, which expressed endogenous Esrrb, overexpression of Esrrb in DU145 cells raised the Esrrb protein concentration to a comparable physiological concentration (Fig. 1b). In addition, our RT-PCR results and RNA-seq results confirmed the estrogen related receptor gamma (Esrrg) was not expressed in DU145 cells. The absence of Esrrg eliminated any possible functional contamination by Esrrg in our Esrrb studies (Fig. 1c).

### Esrrb expression alters mRNA profile

**Fig. 2:**
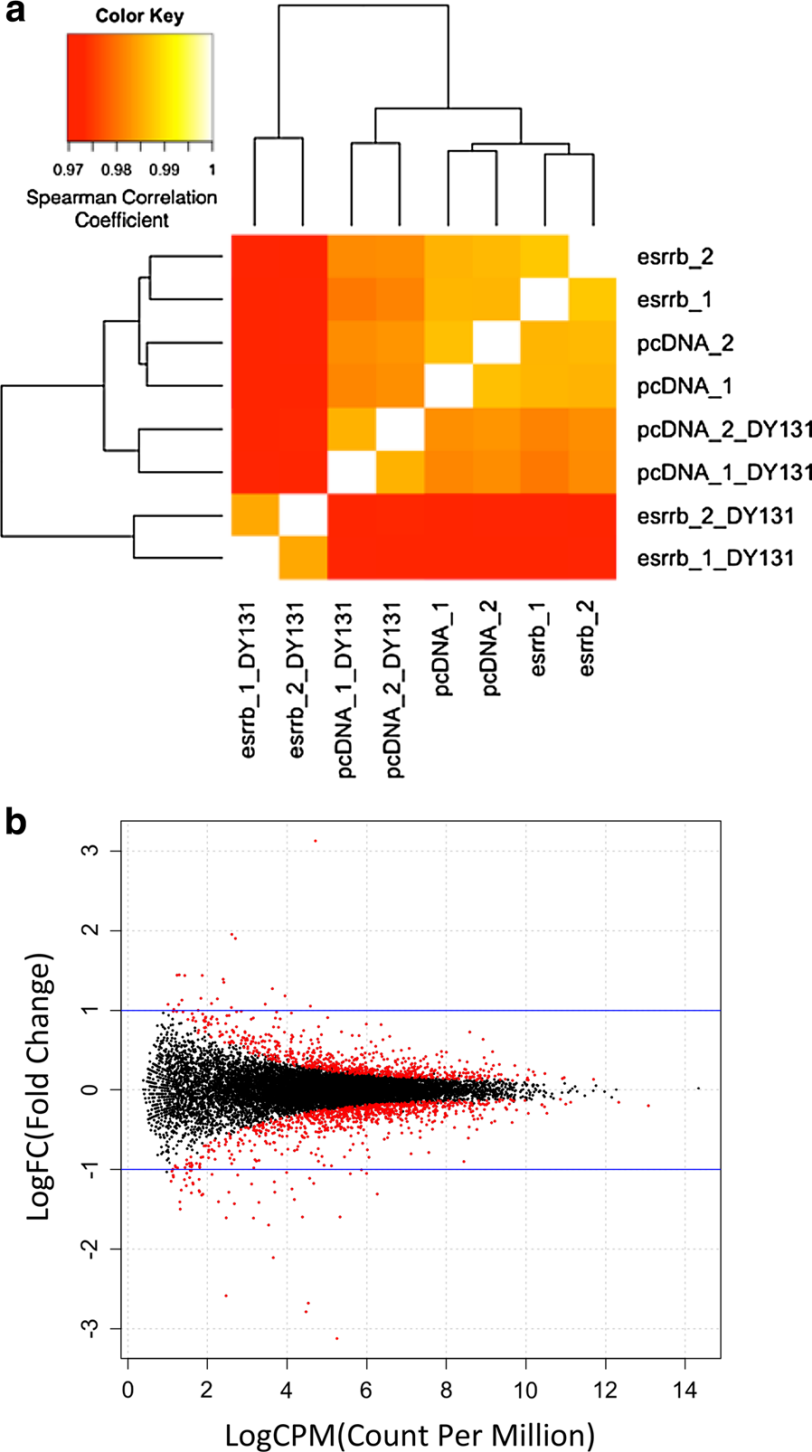
Transcriptome correlation and Esrrb altered mRNAs. **a** Transcriptome correlation analysis was performed using Spearman Ranking Correlation. *Color* represents the correlation coefficient. DY131 treatment to DU145-Esrrb cells results in the lowest correlation coefficient with DU145-pc3.1 cells. **b** *Dot plot* of Esrrb-induced gene expression alteration. Genes expressed at adequate level are tested for differential gene expression test. The *plot* was made by plotting the Log2FC (fold change) against the Log2 cpm (count-per-million) difference. *Red color* marks the genes that are significant differentially expressed (FDR < 0.05), and the *blue lines* marked the Log2FC cutoff value (Log2FC > 1 or Log2FC < −1). 67 genes passed both thresholds

**Fig. 3:**
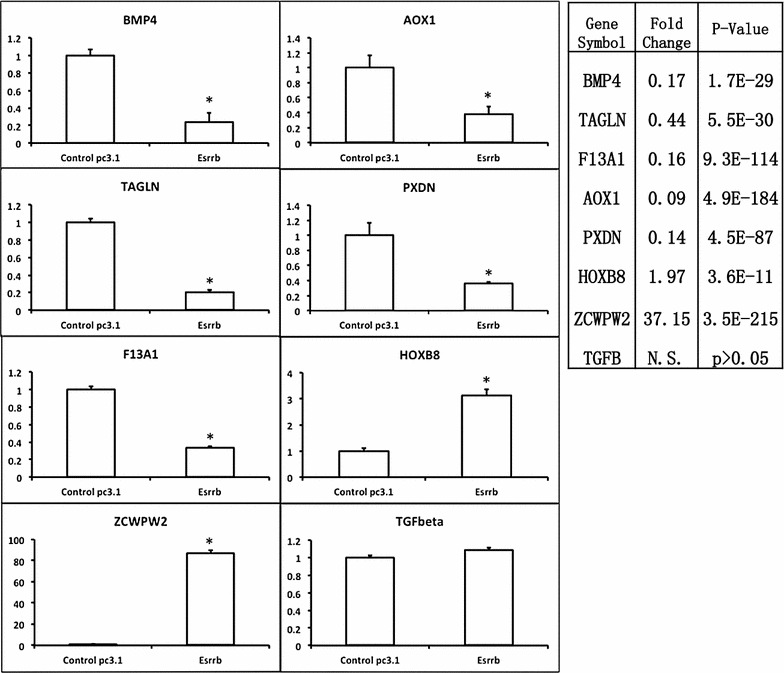
Esrrb-regulated mRNA validation. *Left panel* qPCR validation of Esrrb-regulated mRNAs. Expression values were firstly normalized to Gapdh and normalized ratios are further normalized to that of DU145-pc3.1. *Error bars* represent standard deviation. Student t test was performed for statistical analysis (*p < 0.05). Seven genes were differentially expressed in both RNA-seq and qPCR, 1 gene, TGFbeta, is not differentially expressed in either assay and serves as a negative control. *Right panel* RNA-Seq analysis result, fold change (FC) indicates the ratio of normalized read counts in DU145-Esrrb to that of DU145-pc3.1

To distinguish genes regulated by Esrrb, we performed RNA-Seq analysis on cDNA libraries constructed from two biological replicates of both DU145-pc3.1 and DU145-Esrrb cells. Spearman ranking correlation analysis showed that the expression of Esrrb in DU145 created a distinct transcriptome compared to control DU145-pc3.1 cells (Fig. 2a). We found 67 genes (21 genes up-regulated, 46 genes down-regulated) altered due to Esrrb expression (Fig. 2b; Table 1). Seven genes that are among the most changed genes (*zcwpw2*, *hoxb8*, *tagln*, *f13a1*, *pxdn*, *aox1*, and *bmp4*, as well as *tgfβ* as a negative control) were confirmed by qPCR (Fig. 3). Gene ontology (GO) analysis shows that the products of Esrrb driven differentially expressed genes fell into functional categories of regulation of cell development as well as immune responses (Table 2).

**Table 1:** Esrrb altered mRNAs

Gene symbol	logFC	P value	FDR
AOX1	−3.49	4.89E−184	3.19E−180
PXDN	−2.79	4.45E−87	1.16E−83
F13A1	−2.68	9.28E−114	4.04E−110
BMP4	−2.59	1.69E−29	1.16E−26
NPTX1	−2.11	1.06E−46	1.73E−43
SPNS2	−1.70	8.70E−31	7.09E−28
DDX60	−1.61	2.43E−15	4.46E−13
NEFL	−1.61	1.84E−13	2.85E−11
OASL	−1.60	1.30E−25	7.07E−23
IFIT3	−1.60	4.29E−42	5.09E−39
WDR52	−1.50	1.85E−06	7.40E−05
C3	−1.43	7.81E−09	5.73E−07
LOC344887	−1.41	5.01E−20	1.49E−17
PCDHB15	−1.41	6.25E−06	0.00020978
CXorf57	−1.41	1.78E−10	1.92E−08
IFI6	−1.38	1.09E−19	2.91E−17
CXCR4	−1.32	3.23E−06	0.00011537
GBP1	−1.31	1.43E−07	7.57E−06
IGFBP3	−1.31	3.19E−89	1.04E−85
ZSCAN12P1	−1.30	0.00015585	0.00302695
RNF128	−1.29	1.58E−10	1.73E−08
SAMD9	−1.29	2.69E−22	1.13E−19
UNC5A	−1.28	8.28E−07	3.62E−05
MX2	−1.27	1.68E−06	6.86E−05
SSBP2	−1.26	5.96E−05	0.00141054
MX1	−1.25	5.99E−22	2.37E−19
SULT4A1	−1.20	4.94E−05	0.00120711
DPYD	−1.19	1.35E−05	0.00040257
NEBL	−1.18	1.16E−12	1.62E−10
TAGLN	−1.18	5.48E−30	3.97E−27
INA	−1.17	2.47E−05	0.0006739
BMF	−1.16	3.32E−05	0.00085559
ESRP1	−1.13	0.00014206	0.00283503
GJA3	−1.12	1.38E−05	0.00040614
IFIT2	−1.11	1.02E−19	2.76E−17
LOC1005065	−1.10	8.53E−05	0.0018618
RARRES3	−1.07	1.84E−07	9.68E−06
TMEM45A	−1.06	4.69E−06	0.00016245
LGALS3BP	−1.05	4.57E−17	9.63E−15
ERAP2	−1.05	1.83E−50	3.41E−47
WNT10A	−1.05	0.00024417	0.00440189
PADI2	−1.04	1.13E−17	2.55E−15
REEP1	−1.01	0.00024704	0.0044413
AMIGO2	−1.01	8.26E−45	1.08E−41
HES1	−0.98	1.30E−07	7.01E−06
FRMD4B	−0.98	0.00014442	0.00284746
NRIP3	0.97	4.28E−14	6.90E−12
HOXB8	0.98	3.60E−11	4.33E−09
KCNQ5	0.98	5.87E−08	3.55E−06
COX6B2	0.99	1.24E−07	6.77E−06
PPFIBP2	1.04	6.38E−07	2.92E−05
KIAA1199	1.06	1.21E−25	6.89E−23
BST1	1.08	4.97E−13	7.30E−11
LOC1001336	1.13	0.00019921	0.00370382
SMOC1	1.15	1.52E−06	6.32E−05
LOC441454	1.15	9.40E−06	0.00029713
DDIT4L	1.18	2.28E−10	2.39E−08
SEMA3F	1.27	2.08E−20	6.96E−18
DUOX1	1.35	5.37E−09	4.25E−07
ARHGAP24	1.39	6.40E−10	6.01E−08
CDHR1	1.44	2.04E−06	7.93E−05
SDC2	1.44	1.49E−08	1.04E−06
SH3RF3	1.44	1.32E−05	0.00039562
PRSS8	1.45	6.96E−06	0.00022989
TKTL1	1.90	3.68E−21	1.33E−18
FGB	1.96	5.57E−22	2.27E−19
ZCWPW2	5.22	3.47E−215	4.53E−211

**Table 2:** Gene ontology analysis of Esrrb altered mRNAs

Term	Count	P value
GO:0060284 ~ regulation of cell development	5	0.006
GO:0006955 ~ immune response	8	0.012
GO:0009611 ~ response to wounding	7	0.012
GO:0042542 ~ response to hydrogen peroxide	3	0.017
GO:0050767 ~ regulation of neurogenesis	4	0.022
GO:0006800 ~ oxygen and reactive oxygen species metabolic process	3	0.024
GO:0060052 ~ neurofilament cytoskeleton organization	2	0.029
GO:0000302 ~ response to reactive oxygen species	3	0.03
GO:0051960 ~ regulation of nervous system development	4	0.032
GO:0031960 ~ response to corticosteroid stimulus	3	0.038
GO:0010035 ~ response to inorganic substance	4	0.038
GO:0045661 ~ regulation of myoblast differentiation	2	0.039
GO:0048667 ~ cell morphogenesis involved in neuron differentiation	4	0.04

### DY131 requires Esrrb to affect gene expression

**Fig. 4:**
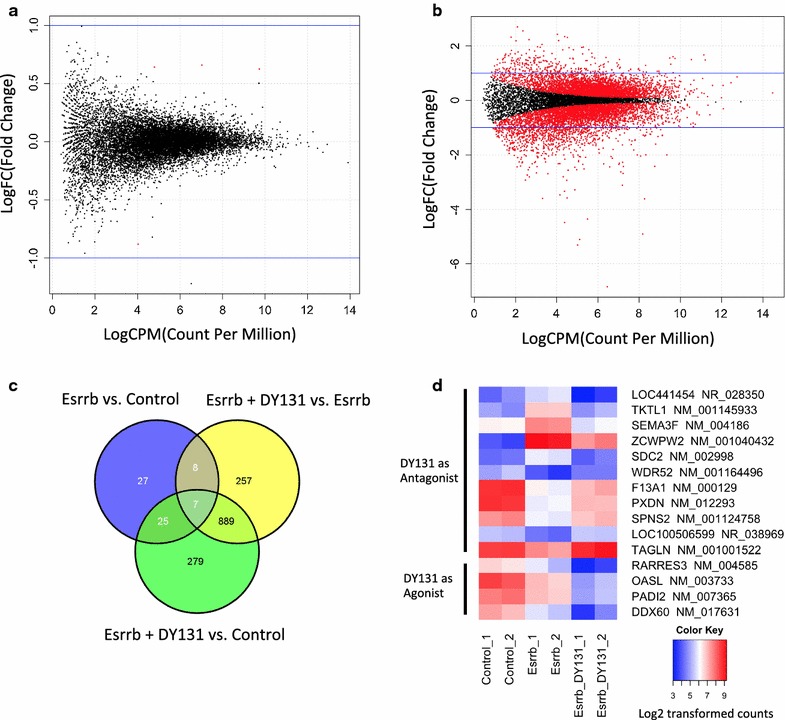
mRNA alteration by DY131 requires Esrrb expression. **a** DY131 treatment alone did not alter the expression of any gene. In contrast, when Esrrb was expressed, **b** DY131 altered 1161 mRNAs. **c** Venn Diagram of pairwise comparisons of altered mRNAs showed 15 (p = 0.0014) Esrrb altered mRNAs can be further regulated by DY131 treatment (overlap between Esrrb vs. control and Esrrb + DY131 vs. Esrrb). **d** Heat map of mRNA concentration of the 15 genes that response to both Esrrb expression as well as DY131 treatment. Log2-transformed normalized read counts of these 15 genes were color coded. DY131 is an agonist for 4 mRNAs that are responsive to Esrrb, while it is an antagonist of Esrrb in regulating the other 11 mRNAs

To get a more comprehensive understanding of Esrrb-regulated genes and characterize Esrrb’s potential ligand dependent activity, control DU145-pc3.1 and DU145-Esrrb cells were treated with Esrrb/Esrrg synthetic ligand DY131. Since both qPCR and RNA-seq show Esrrb transcript concentration is extremely low in DU145 cells and Esrrg is absent, and Esrrb protein concentration is also below the detection limit of western-blot analysis, it was not surprising to observe DY131 treatment without Esrrb expressed did not result in any gene differentially expressed (Fig. 4a). After we applied DY131 to DU145-Esrrb cells, we found DY131 treatment most significantly modified the transcriptome (Figs. 2a, 4b). Further comparison of DU145-Esrrb cells alone to DY131-treated DU145-Esrrb cells detected 1161 altered mRNAs (861 down-regulated, 300 up-regulated). 15 of them overlapped with Esrrb-induced mRNA alterations (Fig. 4c, d; Table 3). We defined an Esrrb agonist as a ligand that moves the mRNA concentration in the direction as Esrrb does; and an antagonist moves the concentration in the opposite direction as Esrrb does. By comparing the trend of the altered genes induced by Esrrb expression and DY131 treatment, DY131 acts as an agonist for 4 of the 15 genes, and an antagonist for 11 of the 15 genes (Fig. 4d). There are another 1146 mRNAs changed with both Esrrb and DY131 treatment compared to Esrrb alone, indicating their responses is ligand-dependent (Table 3).

**Table 3:** DY131 altered mRNAs when Esrrb is expressed

Gene symbol	logFC	P value	FDR
MTRNR2L8	−6.83	0	0
MTRNR2L10	−5.31	9.85E−242	4.62E−239
SNHG5	−5.11	1.21E−277	6.66E−275
RPS29	−4.91	0	0
SEC61G	−4.48	2.94E−165	7.90E−163
RPL36A	−4.35	2.3501596881585	0.00E+00
RPL12	−3.62	0	0
TMEM212	−3.60	1.12E−56	4.20E−55
ESRG	−3.58	6.76E−114	9.61E−112
NEDD8-MDP1	−3.47	7.24E−102	7.97E−100
FOS	−3.34	2.98E−53	9.94E−52
SLIRP	−3.28	1.01E−127	1.85E−125
MTRNR2L1	−3.12	0	0
LGALS3	−2.94	3.19E−115	4.69E−113
SYTL1	−2.93	9.57E−29	1.23E−27
CLIC3	−2.80	7.42E−29	9.60E−28
WISP2	−2.80	7.36E−56	2.71E−54
ABCD3	−2.74	5.71E−21	4.79E−20
TNNT1	−2.69	4.26E−53	1.42E−51
RPL31	−2.68	0	0
ASS1	−2.66	2.78E−30	3.82E−29
EGR1	−2.63	0	0
PSMA1	−2.57	3.99E−89	3.55E−87
MRPL4	−2.56	5.21E−98	5.36E−96
ACPI	−2.55	8.68E−27	1.02E−25
USMG5	−2.54	4.08E−124	6.97E−122
CRIP1	−2.52	7.63E−155	1.76E−152
STAT4	−2.46	5.11E−22	4.64E−21
NDUFA11	−2.45	2.16E−93	2.05E−91
SCFD1	−2.44	6.21E−304	4.14E−301
CYBA	−2.44	5.78E−17	3.76E−16
NAPSA	−2.43	2.66E−17	1.77E−16
RPL13AP5	−2.43	0	0
RPL9	−2.42	0	0
TCTEX1D2	−2.41	6.26E−19	4.62E−18
MKNK2	−2.35	4.60E−83	3.57E−81
RPL18	−2.35	0	0
MUC1	−2.33	2.97E−73	1.75E−71
SEPP1	−2.33	4.57E−14	2.42E−13
PDE9A	−2.32	1.44E−65	7.05E−64
SEMA6B	−2.32	1.67E−53	5.59E−52
RPS15A	−2.30	0	0
CNTN1	−2.29	3.68E−18	2.58E−17
CA11	−2.28	8.82E−26	9.90E−25
TXN	−2.27	0	0
LOC728730	−2.26	4.28E−15	2.45E−14
GSTM1	−2.26	3.08E−39	6.17E−38
MACROD1	−2.26	5.25E−17	3.42E−16
CBLC	−2.25	5.56E−23	5.30E−22
SUCLG2	−2.24	7.28E−112	9.70E−110
PDCD4	−2.21	4.54E−103	5.13E−101
NDUFB2	−2.16	8.58E−73	4.91E−71
C4orf48	−2.16	1.10E−16	7.00E−16
PIR-FIGF	−2.15	1.69E−35	2.92E−34
DHRS3	−2.13	4.91E−20	3.92E−19
RPL38	−2.12	4.53E−247	2.20E−244
COX17	−2.11	5.14E−26	5.86E−25
NFE2	−2.10	7.00E−10	2.63E−09
RAB26	−2.09	1.66E−09	6.04E−09
NAPRT1	−2.09	1.48E−116	2.20E−114
CDK5	−2.08	1.31E−28	1.67E−27
NUCB2	−2.08	2.91E−88	2.49E−86
CEBPD	−2.07	5.29E−83	4.08E−81
DYNC2LI1	−2.07	5.50E−21	4.63E−20
RPL34	−2.06	6.26E−174	1.88E−171
SNRPF	−2.06	3.85E−103	4.39E−101
BCKDHB	−2.05	9.69E−26	1.09E−24
ANXA1	−2.04	0	0
RARS2	−2.04	4.40E−37	8.15E−36
CYP4F11	−2.04	1.11E−18	8.08E−18
SPC24	−2.03	1.42E−17	9.58E−17
PTP4A3	−2.02	4.47E−19	3.34E−18
LOC728190	−2.02	1.35E−12	6.39E−12
PSME1	−2.01	1.62E−81	1.20E−79
POLE2	−2.00	3.59E−57	1.37E−55
NSMCE4A	−2.00	1.78E−61	7.54E−60
FRA10AC1	−2.00	6.45E−43	1.50E−41
RPS12	−2.00	2.44E−295	1.47E−292
MTRNR2L2	−2.00	4.85E−167	1.33E−164
TYMP	−2.00	1.58E−35	2.74E−34
RPL11	−2.00	0	0
CCDC152	−1.99	6.61E−10	2.49E−09
MXD3	−1.96	3.65E−27	4.38E−26
IL17RC	−1.95	3.51E−23	3.39E−22
GRB7	−1.95	1.64E−32	2.49E−31
LOC441454	−1.94	8.75E−10	3.24E−09
PCCA	−1.94	1.00E−21	8.91E−21
ACBD4	−1.94	2.15E−40	4.51E−39
APLP1	−1.93	7.74E−24	7.77E−23
QARS	−1.93	2.90E−156	6.93E−154
STX8	−1.93	7.69E−26	8.67E−25
TM2D1	−1.92	8.60E−25	9.14E−24
C17orf61	−1.92	1.03E−53	3.49E−52
LOC100507156	−1.91	2.19E−24	2.27E−23
KCNAB2	−1.89	1.65E−43	3.93E−42
CERS4	−1.88	1.66E−27	2.01E−26
C18orf8	−1.86	1.06E−73	6.36E−72
NOTCH3	−1.86	1.46E−16	9.26E−16
LHPP	−1.86	1.93E−12	9.03E−12
GNG7	−1.85	2.37E−13	1.19E−12
RTN2	−1.85	2.10E−09	7.55E−09
SEMA3B	−1.84	2.45E−16	1.52E−15
IL6	−1.84	5.35E−17	3.49E−16
LOC644961	−1.84	1.43E−11	6.24E−11
JPX	−1.83	7.74E−11	3.17E−10
CHCHD6	−1.83	7.00E−19	5.14E−18
PNPLA6	−1.83	3.33E−86	2.77E−84
FBLN1	−1.82	5.31E−33	8.33E−32
SIDT2	−1.81	5.19E−40	1.07E−38
DEPTOR	−1.81	1.85E−10	7.26E−10
ZNF826P	−1.81	1.25E−08	4.21E−08
TTC39A	−1.81	4.87E−17	3.18E−16
TM7SF2	−1.81	1.91E−67	9.80E−66
ELMO3	−1.81	2.54E−22	2.33E−21
OOEP	−1.81	8.06E−10	3.00E−09
DNAJC17	−1.81	1.40E−14	7.72E−14
TCEA2	−1.80	1.83E−36	3.29E−35
SLC22A18	−1.80	1.89E−19	1.45E−18
ALDH3B1	−1.80	3.28E−68	1.70E−66
LOC100130872	−1.80	6.08E−10	2.30E−09
ETFA	−1.79	1.34E−136	2.74E−134
THYN1	−1.79	9.07E−41	1.92E−39
AKR1C3	−1.78	1.37E−10	5.47E−10
MT1F	−1.78	1.45E−08	4.86E−08
PIP5KL1	−1.78	2.53E−10	9.85E−10
ATP5D	−1.78	1.68E−44	4.13E−43
TMEM120A	−1.77	1.49E−25	1.64E−24
OSBPL5	−1.77	3.00E−09	1.06E−08
TENC1	−1.77	2.65E−18	1.89E−17
EPHX2	−1.76	3.95E−14	2.10E−13
WDR83	−1.76	4.37E−26	4.99E−25
RUVBL2	−1.75	8.63E−147	1.95E−144
KAZALD1	−1.75	6.39E−22	5.77E−21
RPA3	−1.74	6.68E−41	1.42E−39
NOXA1	−1.74	1.23E−09	4.49E−09
TMEM110-MUSTN1	−1.74	5.21E−08	1.65E−07
CACNA2D2	−1.74	8.47E−10	3.14E−09
DICER1-AS1	−1.73	5.04E−10	1.91E−09
ABHD14A-ACY1	−1.73	1.20E−36	2.19E−35
GRAMD2	−1.73	5.14E−08	1.63E−07
PPIH	−1.73	2.97E−19	2.25E−18
STXBP2	−1.73	2.73E−72	1.55E−70
C10orf10	−1.73	3.54E−21	3.02E−20
PLA2G6	−1.73	5.59E−14	2.93E−13
CHEK2	−1.72	1.79E−46	4.82E−45
SPAG4	−1.72	2.30E−15	1.34E−14
COG6	−1.71	9.36E−37	1.71E−35
TBC1D17	−1.71	7.78E−24	7.79E−23
IFT52	−1.71	6.78E−38	1.30E−36
GARNL3	−1.71	7.22E−11	2.96E−10
DLST	−1.70	2.44E−25	2.67E−24
ACSF2	−1.69	3.58E−13	1.77E−12
RYR1	−1.69	4.48E−09	1.57E−08
LOC100134713	−1.68	3.06E−12	1.41E−11
P2RY6	−1.68	7.55E−21	6.27E−20
RARRES3	−1.68	3.75E−07	1.09E−06
C11orf80	−1.67	5.34E−14	2.81E−13
ELF3	−1.67	2.30E−48	6.58E−47
ADCK4	−1.67	2.68E−40	5.60E−39
GLB1L	−1.67	5.33E−14	2.80E−13
LPPR3	−1.67	1.58E−24	1.66E−23
CDK7	−1.67	1.76E−36	3.16E−35
SLC6A3	−1.66	8.34E−08	2.58E−07
ADAM22	−1.66	2.07E−12	9.64E−12
EIF3K	−1.66	1.65E−98	1.73E−96
S100A4	−1.65	7.75E−10	2.89E−09
ANXA6	−1.65	2.84E−85	2.35E−83
IFT140	−1.65	1.33E−25	1.48E−24
PDIA5	−1.65	1.47E−23	1.44E−22
FADS3	−1.65	2.21E−48	6.32E−47
KCNK15	−1.64	4.58E−21	3.89E−20
FKBP10	−1.64	8.94E−41	1.89E−39
ACSS2	−1.64	1.07E−94	1.06E−92
GSTA4	−1.64	2.80E−31	4.04E−30
KCNMB4	−1.64	8.53E−07	2.38E−06
RPLP2	−1.63	3.48E−220	1.52E−217
UROS	−1.63	2.54E−42	5.73E−41
IFT27	−1.63	4.67E−19	3.49E−18
TRAPPC9	−1.63	6.82E−28	8.44E−27
ADAMTSL4	−1.62	5.48E−43	1.28E−41
EIF3E	−1.62	3.34E−248	1.69E−245
ACY1	−1.62	2.83E−30	3.87E−29
MT1X	−1.62	4.27E−24	4.36E−23
LOC643406	−1.61	4.55E−16	2.79E−15
FBXO36	−1.61	1.48E−14	8.12E−14
PACSIN1	−1.61	5.93E−08	1.87E−07
NIT2	−1.61	4.39E−45	1.10E−43
FUZ	−1.60	3.08E−12	1.42E−11
RPL37	−1.60	2.00E−301	1.27E−298
ALDH4A1	−1.60	2.17E−17	1.45E−16
PDCD5	−1.60	6.19E−65	2.92E−63
MFSD3	−1.60	3.51E−31	5.00E−30
EML3	−1.60	1.27E−64	5.92E−63
PSMA3	−1.60	1.93E−96	1.96E−94
TRPT1	−1.59	2.64E−23	2.56E−22
ALDH6A1	−1.59	9.68E−21	8.00E−20
LOC283038	−1.59	3.12E−11	1.32E−10
BDH2	−1.59	1.27E−17	8.59E−17
SDHB	−1.59	1.43E−70	7.78E−69
ARHGEF25	−1.59	1.35E−29	1.79E−28
CYS1	−1.58	4.38E−08	1.40E−07
C8orf45	−1.58	4.42E−08	1.41E−07
GLTSCR2	−1.57	4.94E−46	1.30E−44
JMJD8	−1.57	3.93E−28	4.90E−27
ANKRD24	−1.57	1.17E−07	3.56E−07
SNURF	−1.57	6.25E−68	3.23E−66
SNRPN	−1.57	6.43E−68	3.31E−66
RCN3	−1.57	1.28E−15	7.56E−15
LACTB2	−1.57	2.50E−78	1.75E−76
HSF4	−1.56	1.23E−13	6.27E−13
ENDOV	−1.56	4.24E−09	1.48E−08
CPT1C	−1.55	4.07E−07	1.17E−06
ITFG1	−1.55	1.10E−88	9.66E−87
IL11RA	−1.55	2.61E−11	1.12E−10
FRG1	−1.55	2.08E−08	6.87E−08
CCDC104	−1.54	8.93E−31	1.25E−29
CERS1	−1.54	4.07E−08	1.30E−07
GDF1	−1.54	4.07E−08	1.30E−07
USP4	−1.54	4.05E−13	2.00E−12
PLCD1	−1.54	6.66E−14	3.47E−13
PBXIP1	−1.54	5.25E−37	9.70E−36
NDUFC1	−1.54	1.61E−42	3.66E−41
CEP70	−1.54	5.71E−59	2.29E−57
MFSD4	−1.54	2.40E−07	7.10E−07
ARPC4-TTLL3	−1.53	3.34E−25	3.64E−24
XRCC1	−1.53	7.89E−35	1.33E−33
CLDN4	−1.52	2.47E−20	2.00E−19
VWA5A	−1.52	7.77E−10	2.90E−09
PC	−1.52	1.73E−20	1.41E−19
MMP11	−1.52	1.58E−09	5.74E−09
C9orf84	−1.52	1.04E−28	1.33E−27
SLC37A2	−1.52	1.08E−06	2.99E−06
SUPT3H	−1.52	5.13E−12	2.33E−11
SLC44A3	−1.52	1.17E−11	5.15E−11
UNC93B1	−1.52	6.48E−37	1.19E−35
SLC38A6	−1.52	1.32E−13	6.69E−13
NDUFA1	−1.51	4.88E−40	1.01E−38
TMEM205	−1.51	4.08E−37	7.58E−36
ZCRB1	−1.51	2.46E−88	2.13E−86
BRSK1	−1.50	7.65E−10	2.86E−09
HDAC5	−1.50	3.47E−33	5.53E−32
RNASEH2B	−1.50	1.90E−22	1.76E−21
CLASRP	−1.50	4.14E−23	3.98E−22
CAMK1	−1.50	1.12E−21	1.00E−20
C11orf10	−1.49	1.26E−42	2.91E−41
PRKCSH	−1.49	5.06E−129	9.41E−127
PMF1-BGLAP	−1.49	3.71E−34	6.12E−33
NFASC	−1.49	1.16E−05	2.86E−05
LTBP4	−1.49	2.21E−61	9.29E−60
LAMA5	−1.49	2.31E−160	6.08E−158
LRSAM1	−1.49	5.65E−26	6.42E−25
CTSH	−1.48	6.14E−31	8.67E−30
HEXB	−1.48	7.22E−108	8.96E−106
MGST2	−1.48	1.97E−09	7.13E−09
FAH	−1.48	8.07E−22	7.23E−21
PEX7	−1.48	2.91E−14	1.56E−13
C5	−1.48	1.05E−11	4.64E−11
ACADS	−1.48	2.02E−21	1.76E−20
IFT43	−1.47	9.38E−15	5.24E−14
JAK3	−1.47	6.46E−12	2.90E−11
MRPL39	−1.47	1.89E−45	4.88E−44
SLC43A1	−1.47	3.84E−08	1.23E−07
EFEMP2	−1.47	9.20E−07	2.56E−06
SSBP4	−1.47	1.48E−14	8.14E−14
IMMP1L	−1.47	1.82E−07	5.46E−07
GPR108	−1.47	1.33E−60	5.50E−59
WDR54	−1.46	4.01E−38	7.75E−37
ARHGAP8	−1.46	4.37E−14	2.32E−13
RPL35A	−1.46	8.72E−183	2.76E−180
GBP2	−1.46	7.43E−12	3.31E−11
TECR	−1.46	1.15E−73	6.86E−72
AUH	−1.46	1.97E−08	6.54E−08
PRR5-ARHGAP8	−1.46	1.99E−14	1.08E−13
LINC00263	−1.45	7.89E−17	5.07E−16
PDLIM2	−1.45	2.48E−44	6.06E−43
RASA2	−1.45	1.41E−19	1.09E−18
PTPN6	−1.45	1.25E−36	2.27E−35
PARL	−1.45	2.07E−65	1.00E−63
CERCAM	−1.45	1.71E−74	1.04E−72
RPL37A	−1.45	3.14E−201	1.14E−198
ARRDC3	−1.45	2.82E−84	2.26E−82
NAE1	−1.45	1.38E−78	9.72E−77
MYZAP	−1.45	7.55E−09	2.60E−08
FBXO2	−1.44	3.57E−23	3.45E−22
C1QL1	−1.44	1.45E−18	1.05E−17
FDPS	−1.44	1.12E−208	4.30E−206
FER1L4	−1.44	2.19E−34	3.63E−33
TMEM8B	−1.44	2.90E−24	3.00E−23
THOC6	−1.44	1.49E−31	2.17E−30
DMPK	−1.43	9.94E−26	1.1 IE−24
RNF181	−1.43	1.88E−42	4.26E−41
GUK1	−1.43	3.52E−94	3.37E−92
GHDC	−1.43	2.52E−15	1.47E−14
GRAMD1A	−1.43	1.76E−70	9.52E−69
SYTL2	−1.43	3.67E−25	3.98E−24
LEPR	−1.43	7.79E−22	7.01E−21
FLJ22184	−1.43	9.59E−18	6.57E−17
EXOSC9	−1.42	5.87E−47	1.59E−45
MMAB	−1.42	2.1 IE−37	4.00E−36
KRT86	−1.42	1.20E−05	2.96E−05
ABHD1	−1.42	7.52E−08	2.34E−07
HOOK2	−1.42	1.59E−22	1.48E−21
PCSK4	−1.42	1.40E−10	5.56E−10
TMC6	−1.42	1.82E−28	2.31E−27
GDPD1	−1.42	6.19E−06	1.57E−05
LRRC23	−1.42	5.41E−11	2.25E−10
PION	−1.42	3.83E−12	1.75E−11
BCL7C	−1.42	9.48E−37	1.73E−35
YPEL3	−1.41	2.79E−10	1.08E−09
RAD51B	−1.41	7.17E−15	4.03E−14
ANXA4	−1.41	3.39E−64	1.55E−62
B4GALNT4	−1.41	8.74E−13	4.18E−12
COX7B	−1.41	1.11E−66	5.56E−65
PRKCZ	−1.41	1.76E−15	1.03E−14
RAB4B	−1.41	2.43E−21	2.10E−20
C4orf34	−1.40	1.44E−38	2.81E−37
STXIO	−1.40	2.42E−15	1.41E−14
CRELD2	−1.40	5.50E−24	5.57E−23
ATP5E	−1.39	6.27E−93	5.87E−91
ESD	−1.39	1.48E−75	9.39E−74
MIA-RAB4B	−1.39	1.84E−21	1.61E−20
NDUFA13	−1.39	5.54E−58	2.17E−56
SNX14	−1.39	3.32E−75	2.09E−73
MRPL13	−1.39	2.86E−44	6.97E−43
PTPRH	−1.39	7.14E−18	4.93E−17
BBS5	−1.39	2.36E−32	3.55E−31
LMBRD1	−1.38	4.17E−25	4.50E−24
IQCH	−1.38	4.75E−08	1.51E−07
LMTK3	−1.38	4.13E−14	2.19E−13
MIR497HG	−1.38	6.24E−12	2.81E−11
C6orf70	−1.38	3.13E−25	3.41E−24
FGGY	−1.38	5.12E−07	1.46E−06
UNC5CL	−1.38	1.66E−08	5.52E−08
DUT	−1.38	8.03E−114	1.13E−111
P2RX4	−1.38	2.17E−20	1.76E−19
HHIPL2	−1.38	4.20E−06	1.09E−05
COX5A	−1.38	3.09E−79	2.21E−77
CKLF	−1.37	6.08E−34	9.94E−33
CRYZL1	−1.37	7.16E−27	8.45E−26
GSTM4	−1.37	6.05E−20	4.80E−19
DNAH14	−1.37	3.44E−11	1.45E−10
TCTN1	−1.37	6.80E−12	3.05E−11
CBX3P2	−1.36	2.01E−06	5.42E−06
PTH1R	−1.36	0.000133911	0.000291689
SEMA6C	−1.36	2.31E−09	8.29E−09
PIR	−1.36	2.10E−31	3.03E−30
DRAP1	−1.36	2.70E−101	2.90E−99
SCP2	−1.36	1.15E−12	5.47E−12
GMDS	−1.36	8.89E−18	6.10E−17
FRG1B	−1.36	9.55E−13	4.56E−12
DECR1	−1.35	4.15E−35	7.08E−34
CTAGE5	−1.35	4.25E−40	8.82E−39
NPM3	−1.35	1.82E−40	3.82E−39
AASS	−1.35	2.75E−05	6.49E−05
ZC3H6	−1.35	1.1 IE−29	1.48E−28
C6orf203	−1.35	2.06E−08	6.82E−08
ADAMTS13	−1.35	1.19E−11	5.23E−11
UBXN11	−1.35	2.01E−06	5.42E−06
C10orf54	−1.35	3.00E−63	1.32E−61
LSS	−1.35	7.02E−41	1.49E−39
KLC4	−1.35	7.74E−09	2.66E−08
ITGB3BP	−1.35	6.76E−11	2.78E−10
TKTL1	−1.35	2.72E−12	1.26E−11
C10orf55	−1.34	1.16E−63	5.16E−62
CRELD1	−1.34	1.99E−20	1.62E−19
ADSSL1	−1.34	2.20E−21	1.91E−20
ALKBH7	−1.34	1.62E−27	1.97E−26
AIFM3	−1.34	5.13E−07	1.47E−06
LLGL2	−1.34	2.44E−09	8.76E−09
SLC27A1	−1.34	1.99E−13	9.99E−13
ZBTB8OS	−1.34	5.50E−20	4.37E−19
ANKRD13D	−1.34	7.36E−25	7.86E−24
C6orf57	−1.34	3.31E−07	9.64E−07
GCAT	−1.33	8.16E−10	3.04E−09
TEX9	−1.33	1.32E−05	3.23E−05
MAP2K5	−1.33	9.00E−11	3.66E−10
SLC27A2	−1.33	2.27E−21	1.97E−20
LTBP3	−1.33	2.53E−43	5.95E−42
LOC100287559	−1.33	4.09E−05	9.47E−05
IFITM10	−1.33	9.64E−09	3.28E−08
CRYL1	−1.33	6.22E−10	2.34E−09
USH1C	−1.33	4.48E−09	1.57E−08
ZC3H12D	−1.33	0.000330901	0.000684453
ERI2	−1.32	7.33E−09	2.53E−08
TBX6	−1.32	0.000899671	0.001760238
WBSCR22	−1.32	1.53E−55	5.53E−54
GNB2L1	−1.32	2.65E−272	1.40E−269
LOC100131089	−1.32	3.24E−08	1.05E−07
EGFL7	−1.32	8.28E−37	1.52E−35
PIM3	−1.31	6.31E−67	3.18E−65
NUCB1	−1.31	1.86E−79	1.34E−77
FDXR	−1.31	1.08E−19	8.38E−19
EMID1	−1.31	2.72E−09	9.72E−09
PIBF1	−1.31	2.54E−16	1.58E−15
HIBCH	−1.30	1.13E−15	6.72E−15
RPS7	−1.30	4.23E−158	1.05E−155
BIK	−1.30	5.76E−06	1.47E−05
TCP11L2	−1.30	9.98E−07	2.77E−06
TSNAX-DISC1	−1.30	6.33E−22	5.72E−21
OMA1	−1.30	1.35E−25	1.50E−24
LOC100506990	−1.30	3.51E−09	1.24E−08
TSTD1	−1.30	7.46E−28	9.23E−27
KISS1R	−1.30	0.000386067	0.000792495
BCKDHA	−1.29	2.10E−43	4.98E−42
B9D1	−1.29	3.14E−13	1.56E−12
ZNF695	−1.29	8.45E−10	3.14E−09
TMEM63B	−1.29	2.19E−69	1.17E−67
MOSPD3	−1.29	8.70E−19	6.37E−18
RNASE4	−1.29	2.00E−27	2.42E−26
UGGT2	−1.29	6.04E−21	5.06E−20
SEMA3F	−1.29	2.32E−19	1.77E−18
RPS24	−1.29	6.92E−214	2.83E−211
DAK	−1.29	4.77E−62	2.04E−60
LOC100130691	−1.29	0.000198722	0.0004236
CTU2	−1.29	1.68E−13	8.48E−13
PLD3	−1.29	7.26E−45	1.81E−43
RHOV	−1.29	2.06E−15	1.20E−14
CHPT1	−1.29	5.19E−67	2.64E−65
ACSM3	−1.29	3.03E−11	1.29E−10
RPS25	−1.28	4.03E−185	1.31E−182
OASL	−1.28	2.48E−11	1.06E−10
RPLP1	−1.28	7.23E−219	3.05E−216
C19orf79	−1.28	2.43E−11	1.04E−10
IL20RB	−1.28	1.18E−09	4.32E−09
CACNG6	−1.28	1.23E−05	3.02E−05
TBCE	−1.28	1.40E−28	1.78E−27
FBXO16	−1.28	0.00019181	0.000409623
LOC100505549	−1.28	9.29E−06	2.31E−05
LOC100507218	−1.28	0.000213956	0.000453549
TLR5	−1.28	0.000112273	0.000247109
EML2	−1.28	1.54E−14	8.42E−14
NPM1	−1.28	3.12E−138	6.48E−136
GAA	−1.28	1.30E−32	1.99E−31
NKD2	−1.28	4.23E−10	1.61E−09
CRIP2	−1.28	8.19E−11	3.34E−10
LOC100132891	−1.27	6.71E−07	1.89E−06
ALG5	−1.27	2.16E−16	1.35E−15
PXK	−1.27	1.02E−14	5.67E−14
ADA	−1.27	6.1 IE−14	3.20E−13
GALE	−1.27	3.38E−45	8.55E−44
PHGDH	−1.27	3.96E−113	5.44E−111
CREG2	−1.27	0.000426556	0.000871508
MSLN	−1.27	2.53E−19	1.93E−18
GDPD5	−1.27	1.22E−12	5.80E−12
ITGA7	−1.27	5.07E−06	1.30E−05
LIG1	−1.27	1.36E−27	1.66E−26
LRTOMT	−1.26	4.37E−06	1.13E−05
C17orf49	−1.26	1.37E−44	3.39E−43
HMGN5	−1.26	5.93E−07	1.69E−06
LOC100505624	−1.26	4.04E−10	1.55E−09
CATSPER1	−1.25	0.000949224	0.00184719
TLE2	−1.25	2.35E−16	1.46E−15
CES3	−1.25	5.98E−07	1.70E−06
TTC35	−1.25	7.52E−30	1.01E−28
C6orf72	−1.25	2.65E−33	4.23E−32
RPS19	−1.25	1.01E−168	2.84E−166
EVI5L	−1.25	3.82E−23	3.68E−22
LOC81691	−1.25	7.76E−11	3.17E−10
PLEKHH3	−1.25	5.91E−39	1.17E−37
LOC100507501	−1.24	1.34E−06	3.67E−06
SLC25A5-AS1	−1.24	2.22E−20	1.80E−19
RPS8	−1.24	1.41E−201	5.25E−199
PPP1R7	−1.24	3.54E−37	6.58E−36
MAGED2	−1.24	4.04E−41	8.64E−40
CSTF3	−1.24	1.10E−12	5.24E−12
LINC00467	−1.24	1.39E−17	9.42E−17
MAD2L2	−1.24	4.26E−29	5.54E−28
PCCB	−1.24	2.05E−53	6.87E−52
SEZ6L2	−1.24	1.72E−27	2.09E−26
FKBP2	−1.24	8.99E−30	1.20E−28
DOCK6	−1.24	4.80E−35	8.13E−34
WIPI1	−1.24	2.07E−06	5.57E−06
ECH1	−1.23	5.12E−76	3.32E−74
OCEL1	−1.23	7.19E−14	3.74E−13
ZNF385C	−1.23	2.37E−06	6.31E−06
ATP8B3	−1.23	6.42E−24	6.47E−23
PAFAH1B2	−1.23	8.05E−25	8.57E−24
TM4SF19-TCTEX1D	−1.23	1.23E−05	3.02E−05
SRPX	−1.23	5.84E−11	2.42E−10
SLC39A11	−1.23	3.03E−21	2.60E−20
TMEM41B	−1.22	5.82E−09	2.02E−08
PADI2	−1.22	3.51E−11	1.48E−10
STX4	−1.22	8.39E−28	1.03E−26
MAP4K2	−1.22	1.86E−20	1.51E−19
PXMP4	−1.22	2.50E−07	7.39E−07
TCIRG1	−1.22	2.38E−41	5.15E−40
SERPING1	−1.22	8.23E−08	2.55E−07
IFI35	−1.22	4.92E−13	2.41E−12
DPY19L1P1	−1.22	3.99E−07	1.15E−06
MAN2B1	−1.22	5.94E−48	1.67E−46
FAF1	−1.22	5.28E−51	1.65E−49
ZDHHC1	−1.22	2.34E−06	6.25E−06
NAAA	−1.21	4.63E−11	1.93E−10
EFCAB11	−1.21	4.21E−05	9.73E−05
HSCB	−1.21	1.67E−12	7.85E−12
FBXW9	−1.21	2.21E−05	5.28E−05
ZNF467	−1.21	1.19E−09	4.36E−09
ILVBL	−1.21	1.32E−44	3.28E−43
SDR16C5	−1.21	4.72E−17	3.09E−16
IQGAP2	−1.21	6.21E−07	1.76E−06
SRGAP3	−1.21	2.76E−05	6.50E−05
EGF	−1.21	0.000108319	0.000238866
ERGIC3	−1.21	2.97E−63	1.31E−61
CYFIP2	−1.21	3.35E−14	1.79E−13
BCAS3	−1.21	5.34E−23	5.11E−22
DOCK11	−1.21	3.15E−11	1.33E−10
SLC37A1	−1.21	1.67E−07	5.02E−07
HSD17B4	−1.21	1.16E−54	4.10E−53
NT5M	−1.21	7.71E−05	0.000172918
SERINC5	−1.20	2.82E−06	7.46E−06
CCDC85B	−1.20	9.58E−95	9.54E−93
ALDH7A1	−1.20	1.18E−61	5.00E−60
OPLAH	−1.20	1.60E−21	1.41E−20
ASNS	−1.20	4.29E−106	5.17E−104
KIFAP3	−1.20	4.03E−26	4.60E−25
C1R	−1.20	2.83E−07	8.29E−07
FRY	−1.20	0.000920901	0.001797881
ANO9	−1.20	7.19E−07	2.02E−06
BCAM	−1.20	7.19E−33	1.1 IE−31
MED30	−1.20	1.06E−13	5.46E−13
LOC100127983	−1.20	6.85E−06	1.73E−05
CBS	−1.20	1.10E−55	4.00E−54
PNPLA2	−1.19	7.09E−37	1.30E−35
C1QL4	−1.19	1.08E−07	3.32E−07
LOC100129716	−1.19	0.000363791	0.000749078
ANKRD36BP2	−1.19	0.000287863	0.000599766
LCMT1	−1.19	7.16E−13	3.45E−12
SHF	−1.19	3.46E−06	9.04E−06
RABGGTA	−1.19	2.76E−10	1.07E−09
ANKRA2	−1.19	4.84E−15	2.76E−14
SYT12	−1.19	8.47E−20	6.64E−19
PYROXD2	−1.19	2.34E−06	6.25E−06
COPG2	−1.19	2.42E−35	4.16E−34
RAP1GAP	−1.19	1.26E−08	4.24E−08
LOC728743	−1.18	2.13E−05	5.11E−05
SRI	−1.18	5.42E−43	1.27E−41
DDX43	−1.18	2.82E−23	2.74E−22
PRIM1	−1.18	1.39E−29	1.85E−28
FAM125A	−1.18	2.67E−16	1.65E−15
HCFC1R1	−1.18	6.80E−52	2.20E−50
THBS3	−1.18	3.57E−18	2.51E−17
C15orf48	−1.18	6.58E−30	8.85E−29
C11orf54	−1.18	7.03E−18	4.86E−17
CTSF	−1.18	5.63E−26	6.40E−25
CDH3	−1.18	4.89E−59	1.96E−57
ULK4	−1.18	0.000777011	0.001532576
C1S	−1.17	6.98E−05	0.00015741
VSIG1	−1.17	0.000372512	0.00076579
MED25	−1.17	6.05E−19	4.47E−18
AIG1	−1.17	7.65E−17	4.93E−16
VAV1	−1.17	2.41E−14	1.30E−13
PPA2	−1.17	1.32E−33	2.13E−32
FAM98C	−1.17	5.33E−05	0.000121815
FCGRT	−1.17	1.69E−26	1.95E−25
EXOSC8	−1.17	1.71E−25	1.88E−24
TMEM160	−1.17	1.88E−11	8.15E−11
SREBF1	−1.17	2.51E−32	3.78E−31
Clorf172	−1.17	8.99E−06	2.24E−05
MSI2	−1.17	1.73E−11	7.52E−11
IMPA2	−1.17	6.70E−36	1.17E−34
IGFBP6	−1.17	1.04E−18	7.55E−18
EIF2D	−1.17	7.55E−48	2.12E−46
LTA4H	−1.16	7.60E−76	4.88E−74
ASL	−1.16	7.72E−28	9.53E−27
ETHE1	−1.16	1.72E−23	1.69E−22
RPH3AL	−1.16	4.72E−21	3.99E−20
KLHDC2	−1.16	2.62E−74	1.59E−72
FAM171A2	−1.16	2.02E−09	7.29E−09
IFT88	−1.16	2.41E−07	7.15E−07
SIGIRR	−1.16	1.53E−05	3.72E−05
SUGT1	−1.16	1.60E−19	1.23E−18
TXNIP	−1.16	3.34E−07	9.72E−07
GTF2H2D	−1.16	0.000102722	0.000227077
REEP6	−1.15	1.93E−18	1.38E−17
AAAS	−1.15	5.67E−34	9.29E−33
CDKL2	−1.15	0.000611109	0.001221929
MRC2	−1.15	2.67E−32	4.02E−31
RPN2	−1.15	4.07E−177	1.26E−174
FN3K	−1.15	5.62E−11	2.33E−10
ST14	−1.15	5.82E−11	2.41E−10
GRAPL	−1.15	0.002321122	0.004253204
CUEDC2	−1.15	2.06E−28	2.59E−27
IFI30	−1.15	1.81E−08	6.01E−08
C9orf46	−1.15	1.24E−12	5.89E−12
ABCA5	−1.15	1.85E−07	5.53E−07
RPGR	−1.15	2.48E−07	7.34E−07
PKN1	−1.15	2.23E−84	1.80E−82
ATG16L2	−1.15	1.06E−06	2.95E−06
WBSCR27	−1.15	3.77E−06	9.80E−06
LRRC45	−1.14	1.24E−17	8.40E−17
PTMS	−1.14	1.81E−79	1.31E−77
CKLF-CMTM1	−1.14	1.06E−16	6.72E−16
BTC	−1.14	3.10E−05	7.27E−05
TNFSF12-TNFS”13	−1.14	7.55E−19	5.53E−18
PREX1	−1.14	4.53E−09	1.58E−08
FGD3	−1.14	7.45E−05	0.000167354
PCIF1	−1.14	2.14E−34	3.56E−33
CALB2	−1.14	3.17E−06	8.33E−06
PTGES	−1.14	3.74E−21	3.18E−20
HES7	−1.13	0.003803794	0.00674057
FGFR4	−1.13	8.06E−24	8.07E−23
NFKBID	−1.13	0.00094651	0.001842867
BMP1	−1.13	1.37E−31	2.00E−30
MSI1	−1.13	0.002464428	0.004500152
RPS6KB2	−1.13	2.74E−20	2.21E−19
KCTD19	−1.13	3.06E−06	8.06E−06
CCDC88B	−1.13	1.55E−11	6.73E−11
SCNN1A	−1.13	4.23E−35	7.21E−34
POMGNT1	−1.13	8.85E−41	1.88E−39
HECTD2	−1.13	0.001588305	0.002987874
NUP107	−1.13	2.10E−54	7.31E−53
CXCL16	−1.13	5.14E−12	2.33E−11
GAPDHS	−1.13	0.001914622	0.003556771
CDC42BPG	−1.13	3.79E−08	1.22E−07
MLXIPL	−1.12	6.00E−06	1.53E−05
IFI27L1	−1.12	1.31E−13	6.68E−13
ABCA7	−1.12	5.03E−32	7.44E−31
CREB3L4	−1.12	8.76E−17	5.61E−16
COPE	−1.12	1.68E−31	2.44E−30
PEMT	−1.12	4.08E−08	1.31E−07
PKN3	−1.12	2.05E−12	9.57E−12
UQCRC1	−1.12	3.06E−84	2.42E−82
DNAJC4	−1.12	2.58E−15	1.50E−14
FAM175A	−1.12	3.67E−05	8.54E−05
FIBP	−1.12	1.34E−48	3.86E−47
KCNN1	−1.12	1.14E−05	2.82E−05
RQCD1	−1.12	5.66E−19	4.19E−18
JUNB	−1.11	9.88E−50	2.95E−48
ASPSCR1	−1.11	5.66E−13	2.76E−12
QPCTL	−1.11	4.68E−25	5.04E−24
CD9	−1.11	3.02E−94	2.91E−92
SH2B2	−1.11	6.68E−05	0.000151083
SSR4	−1.11	1.05E−19	8.16E−19
NDUFA2	−1.11	8.18E−19	5.99E−18
ALPK1	−1.11	1.89E−16	1.19E−15
GFM2	−1.11	1.22E−54	4.29E−53
GPCPD1	−1.11	8.79E−19	6.43E−18
NDRG2	−1.11	1.37E−06	3.74E−06
PRSS22	−1.11	0.002080435	0.003844256
MST1P9	−1.11	0.002266527	0.004161499
TRIM9	−1.11	0.000204924	0.000435644
ATP2A3	−1.11	4.09E−09	1.44E−08
TMEM161A	−1.11	2.06E−12	9.63E−12
ING4	−1.11	5.53E−08	1.75E−07
METTL5	−1.11	9.00E−34	1.46E−32
IFT74	−1.11	2.99E−11	1.27E−10
GALT	−1.10	9.23E−14	4.76E−13
ZCWPW2	−1.10	2.10E−32	3.17E−31
USH1G	−1.10	0.002619829	0.004769462
FAM162A	−1.10	1.90E−18	1.36E−17
BCL3	−1.10	3.72E−46	9.84E−45
TSPAN1	−1.10	9.28E−12	4.1 IE−11
SIPA1	−1.10	2.57E−25	2.80E−24
WDR27	−1.10	8.86E−07	2.47E−06
LOC678655	−1.10	2.98E−09	1.06E−08
MATN2	−1.10	3.00E−17	1.98E−16
SERPINI1	−1.10	7.67E−07	2.15E−06
NPRL2	−1.10	8.74E−12	3.88E−11
IRF6	−1.10	3.53E−11	1.49E−10
C17orf57	−1.10	9.25E−06	2.30E−05
HSPB11	−1.10	2.47E−11	1.06E−10
LOXL2	−1.10	2.56E−119	4.1 IE−117
GATS	−1.10	3.88E−07	1.12E−06
POLD1	−1.10	3.61E−38	6.98E−37
KREMEN2	−1.10	0.000906345	0.001771925
SH3YL1	−1.09	7.15E−18	4.93E−17
HEXDC	−1.09	3.33E−08	1.08E−07
CHIC2	−1.09	5.08E−05	0.000116308
FLJ39051	−1.09	0.000444045	0.000904903
ALKBH6	−1.09	4.88E−09	1.70E−08
MAGOH	−1.09	1.09E−14	6.06E−14
LOC100505783	−1.09	1.52E−05	3.70E−05
C16orf62	−1.09	1.35E−24	1.42E−23
GAL3ST1	−1.09	0.004091279	0.00721967
ZNF670-ZNF695	−1.09	6.71E−07	1.89E−06
UGCG	−1.09	2.19E−49	6.44E−48
AS3MT	−1.09	0.000326442	0.000675804
GRTP1	−1.09	2.37E−05	5.64E−05
AQP3	−1.09	3.02E−08	9.82E−08
TMEM45B	−1.09	7.62E−14	3.95E−13
ZP3	−1.09	2.43E−08	7.96E−08
AP4M1	−1.09	8.43E−09	2.89E−08
PLD1	−1.09	2.70E−06	7.14E−06
CCBL2	−1.09	2.76E−20	2.23E−19
NR4A1	−1.08	4.86E−21	4.10E−20
BRE	−1.08	1.97E−33	3.15E−32
PCYOX1L	−1.08	3.06E−15	1.77E−14
KIAA1456	−1.08	4.80E−05	0.000110264
AARS	−1.08	1.30E−192	4.43E−190
MRPL47	−1.08	2.55E−16	1.58E−15
ERP44	−1.08	4.10E−42	9.20E−41
ARHGEF16	−1.08	5.13E−13	2.51E−12
TP53TG1	−1.08	1.83E−05	4.40E−05
FA2H	−1.08	1.77E−08	5.89E−08
ADAM15	−1.07	4.71E−73	2.73E−71
STAG3	−1.07	0.005718352	0.00986248
PTK2B	−1.07	4.07E−14	2.16E−13
NSMCE1	−1.07	5.38E−18	3.74E−17
ATXNIO	−1.07	9.31E−82	6.97E−80
CCDC53	−1.07	3.48E−15	2.01E−14
MIPEP	−1.07	4.92E−10	1.87E−09
TNFAIP2	−1.07	1.57E−146	3.48E−144
PSMA5	−1.07	1.18E−41	2.60E−40
INSIG1	−1.07	8.19E−55	2.90E−53
KIAA1383	−1.07	4.15E−06	1.07E−05
SDC2	−1.07	3.27E−05	7.64E−05
COX5B	−1.07	7.29E−32	1.08E−30
DTX4	−1.07	2.75E−06	7.28E−06
LOC100289495	−1.07	7.59E−05	0.000170296
BIN1	−1.07	8.92E−11	3.62E−10
CLDN7	−1.07	2.52E−17	1.68E−16
LMF1	−1.07	1.18E−05	2.90E−05
C11orf93	−1.07	0.000180253	0.000386051
C1RL	−1.06	2.49E−30	3.42E−29
MTMR11	−1.06	8.82E−20	6.91E−19
CST6	−1.06	4.12E−16	2.53E−15
CRISPLD1	−1.06	1.71E−07	5.16E−07
PFKL	−1.06	3.28E−73	1.92E−71
IER5L	−1.06	1.03E−19	8.04E−19
NUDT17	−1.06	2.65E−07	7.80E−07
RABAC1	−1.06	9.34E−31	1.31E−29
ABCA2	−1.06	2.56E−49	7.50E−48
TRAP1	−1.06	1.61E−76	1.05E−74
BBS9	−1.06	1.98E−11	8.56E−11
MMP15	−1.06	3.72E−27	4.45E−26
SCPEP1	−1.06	4.13E−22	3.76E−21
TLL2	−1.06	4.28E−05	9.90E−05
VPS28	−1.06	3.44E−19	2.60E−18
TCN2	−1.06	9.31E−07	2.59E−06
HS1BP3	−1.06	5.55E−20	4.41E−19
HMG20B	−1.06	9.18E−84	7.22E−82
FUCA1	−1.06	4.76E−18	3.32E−17
ARHGEF7	−1.05	1.63E−11	7.10E−11
WDR33	−1.05	6.18E−10	2.33E−09
SYT13	−1.05	0.004669962	0.008171303
C16orf13	−1.05	3.16E−24	3.26E−23
KTN1	−1.05	6.31E−91	5.75E−89
GPX4	−1.05	4.33E−82	3.26E−80
RPS16	−1.05	2.85E−107	3.46E−105
AGXT2L2	−1.05	1.67E−12	7.88E−12
TMEM141	−1.05	1.52E−18	1.09E−17
LAMP3	−1.05	4.66E−06	1.20E−05
CDKN1C	−1.05	0.000424019	0.000866884
LOC100288846	−1.05	0.000988006	0.001917938
DHRS12	−1.05	0.008237024	0.013823818
ATP6AP1L	−1.05	0.008191315	0.013754406
ERCC2	−1.05	4.13E−23	3.98E−22
FMO5	−1.05	0.008652057	0.014449416
ULK2	−1.05	8.06E−20	6.33E−19
SMARCD3	−1.05	2.06E−08	6.82E−08
PHYHD1	−1.05	4.25E−11	1.78E−10
C10orf11	−1.05	0.003354135	0.006000913
KRTCAP3	−1.05	0.000203547	0.000433154
SRP54	−1.05	2.70E−88	2.32E−86
IMMP2L	−1.04	2.06E−07	6.13E−07
CARS	−1.04	3.50E−44	8.52E−43
RPL24	−1.04	5.98E−76	3.86E−74
GSN	−1.04	3.51E−17	2.31E−16
BAI2	−1.04	9.31E−16	5.58E−15
WDR18	−1.04	1.17E−46	3.16E−45
ZC4H2	−1.04	0.009400904	0.015597066
EIF3M	−1.04	4.78E−59	1.93E−57
SLC25A42	−1.04	7.39E−08	2.30E−07
MTHFR	−1.04	2.62E−10	1.02E−09
ABCG2	−1.04	0.003349706	0.005994684
NR1H3	−1.04	0.000505923	0.001023253
PAAF1	−1.04	2.18E−21	1.90E−20
GSTK1	−1.04	1.65E−37	3.13E−36
DEPDC4	−1.04	0.000125031	0.000273146
ZNF396	−1.04	2.31E−05	5.51E−05
BHLHE40	−1.04	2.30E−66	1.15E−64
TECPR1	−1.04	3.48E−13	1.72E−12
AMN1	−1.04	1.59E−07	4.80E−07
NTPCR	−1.04	3.26E−21	2.79E−20
MVD	−1.04	5.49E−40	1.13E−38
RRAS	−1.04	1.37E−24	1.44E−23
LOC144481	−1.03	0.001555384	0.00293074
SURF1	−1.03	1.48E−25	1.64E−24
MFF	−1.03	1.03E−98	1.09E−96
MAGED1	−1.03	3.74E−72	2.11E−70
TBL3	−1.03	7.98E−25	8.51E−24
DYX1C1	−1.03	0.000142357	0.000309076
SLC16A5	−1.03	2.35E−23	2.30E−22
GPRIN2	−1.03	1.03E−10	4.17E−10
LOC100130015	−1.03	2.39E−07	7.07E−07
DDX60	−1.03	0.000267213	0.000559136
MITD1	−1.03	1.44E−11	6.27E−11
RBP1	−1.03	0.006467371	0.011039757
TBCA	−1.03	5.18E−33	8.15E−32
ICAM5	−1.03	5.23E−14	2.75E−13
TNFRSF10C	−1.03	0.00023491	0.000494816
CPE	−1.03	7.1 IE−14	3.70E−13
ANK2	−1.03	3.67E−11	1.54E−10
C22orf26	−1.03	0.000452354	0.000920647
SNX2	−1.03	6.73E−33	1.04E−31
ANXA3	−1.03	1.06E−78	7.48E−77
C15orf58	−1.02	0.00133034	0.002534258
RBX1	−1.02	1.31E−21	1.16E−20
ABCD1	−1.02	4.71E−08	1.50E−07
P4HA3	−1.02	0.000276213	0.00057711
KRBA2	−1.02	0.004111753	0.007253308
GLS2	−1.02	0.000259768	0.000544639
ENDOG	−1.02	5.93E−08	1.87E−07
COX7C	−1.02	6.09E−67	3.08E−65
C8orf59	−1.02	2.88E−20	2.32E−19
RAB11FIP4	−1.02	6.08E−09	2.11E−08
CDKL1	−1.02	8.11E−06	2.03E−05
LOC100133957	−1.02	0.001919501	0.003564787
DENND1A	−1.02	5.20E−33	8.18E−32
TRAM1	−1.02	1.47E−76	9.69E−75
UPK3B	−1.02	9.22E−07	2.56E−06
ANKRD29	−1.02	1.13E−27	1.38E−26
CHMP5	−1.02	7.09E−56	2.61E−54
CCDC125	−1.02	4.05E−07	1.17E−06
MEF2BNB-MEF2B	−1.01	0.000476068	0.000966579
PTPRE	−1.01	2.62E−47	7.25E−46
MAGIX	−1.01	0.012672093	0.020531485
MDP1	−1.01	0.000316356	0.000656213
SEMA4G	−1.01	2.18E−21	1.90E−20
TRMT11	−1.01	7.49E−10	2.80E−09
TNFRSF9	−1.01	0.001333696	0.002540269
AMZ2P1	−1.01	1.77E−07	5.32E−07
C7orf10	−1.01	0.000709162	0.001406426
PRPF40B	−1.01	5.32E−24	5.39E−23
NCOA7	−1.01	8.97E−40	1.84E−38
MPND	−1.01	0.000182363	0.000390505
C17orf28	−1.01	1.33E−07	4.04E−07
PFDN5	−1.01	1.94E−57	7.44E−56
VWA1	−1.01	1.13E−18	8.22E−18
VPS33B	−1.01	4.79E−11	2.00E−10
PHYHIP	−1.00	2.49E−07	7.35E−07
SUSD2	−1.00	2.55E−29	3.35E−28
CCNA1	−1.00	1.64E−08	5.47E−08
GAMT	−1.00	4.53E−10	1.72E−09
SLC44A2	−1.00	2.72E−41	5.85E−40
ODF2L	−1.00	2.38E−16	1.48E−15
HIST1H1C	−1.00	2.07E−43	4.93E−42
TAF10	−1.00	6.64E−28	8.23E−27
AKT3	−1.00	7.55E−14	3.92E−13
MACROD2	−1.00	0.008868792	0.014782098
ADAM23	−1.00	1.90E−08	6.31E−08
COQ6	−1.00	2.59E−13	1.29E−12
DLEU2	−1.00	0.005572257	0.009628891
CAT	−1.00	1.55E−30	2.14E−29
MSMO1	−1.00	4.14E−33	6.57E−32
LOC100506334	−1.00	3.11E−06	8.17E−06
TARS2	−1.00	2.70E−38	5.25E−37
P4HTM	−1.00	1.17E−15	6.93E−15
EBF4	−1.00	0.001175315	0.002258326
ARHGEF26-AS1	−1.00	0.000118469	0.000259887
OSGEPL1	−1.00	1.23E−11	5.41E−11
PPFIA3	−1.00	2.52E−05	5.96E−05
C19orf71	−1.00	1.03E−05	2.56E−05
CECR2	−1.00	0.000117675	0.000258236
NAT14	−0.99	1.20E−13	6.15E−13
FADS2	−0.99	2.47E−79	1.77E−77
CALCOCOl	−0.99	3.84E−18	2.69E−17
APOL1	−0.99	0.000590629	0.001183409
ITFG3	−0.99	2.53E−39	5.08E−38
KDELC1	−0.99	1.11E−06	3.05E−06
RPL3	−0.99	1.72E−158	4.35E−156
PLCL2	0.99	1.02E−08	3.47E−08
AOC2	0.99	5.20E−07	1.48E−06
ZBTB2	0.99	3.55E−64	1.61E−62
LOC387647	0.99	2.94E−28	3.68E−27
GDF11	0.99	1.84E−42	4.17E−41
LOC100130992	0.99	6.14E−14	3.21E−13
ZNF407	0.99	1.38E−21	1.22E−20
LOC100288615	0.99	2.15E−11	9.26E−11
TEX15	0.99	1.21E−49	3.59E−48
PMS2P5	0.99	2.22E−10	8.68E−10
TSPYL4	1.00	3.02E−31	4.33E−30
FICD	1.00	4.01E−07	1.16E−06
ZNF587	1.00	3.63E−30	4.94E−29
ANKRD50	1.00	4.80E−50	1.46E−48
NR5A2	1.00	3.61E−06	9.42E−06
ZBTB40	1.00	9.91E−30	1.32E−28
SLAMF7	1.00	1.57E−11	6.83E−11
LOC100129046	1.00	2.73E−07	8.03E−07
PHLPP2	1.00	2.77E−38	5.37E−37
ZNF267	1.00	7.57E−20	5.95E−19
FLNC	1.00	9.43E−158	2.29E−155
ZNF850	1.00	1.62E−14	8.85E−14
HOXB6	1.00	7.96E−27	9.37E−26
RNF34	1.00	6.70E−44	1.61E−42
FOXO3	1.01	5.30E−30	7.17E−29
HUS1	1.01	1.05E−20	8.65E−20
ZNF185	1.01	1.74E−82	1.33E−80
GJA1	1.01	6.19E−16	3.75E−15
AP1S2	1.01	1.54E−19	1.19E−18
TUBB2A	1.01	9.33E−18	6.40E−17
IL16	1.01	0.000167472	0.000360202
ZNF799	1.02	1.30E−09	4.76E−09
LOC100505648	1.02	0.000565896	0.001136915
FAM160A1	1.02	0.004606923	0.008071049
KCTD7	1.02	8.21E−16	4.95E−15
ZNF271	1.02	7.41E−78	5.06E−76
LOC401588	1.02	7.86E−08	2.44E−07
EIF5A	1.02	1.72E−190	5.72E−188
ABHD16B	1.02	1.13E−13	5.79E−13
NBPF15	1.03	4.54E−51	1.43E−49
LOC283922	1.03	0.001810781	0.003376264
GDAP1	1.03	2.93E−52	9.56E−51
KANSL1	1.03	2.80E−09	9.97E−09
HOXB5	1.03	4.62E−16	2.83E−15
LRRC37A4P	1.03	4.01E−19	3.00E−18
RCBTB2	1.04	0.003040041	0.005483952
MYB	1.04	7.95E−05	0.000177891
SLC35F3	1.04	4.08E−27	4.87E−26
LOC100287314	1.04	0.00752343	0.012712245
ZNF33A	1.04	7.90E−65	3.71E−63
TARDBP	1.04	2.43E−135	4.87E−133
FLJ42627	1.04	3.98E−10	1.52E−09
ZNF239	1.04	4.56E−32	6.76E−31
FAM86DP	1.04	1.66E−10	6.54E−10
IL1RL1	1.04	1.54E−08	5.17E−08
ZNF655	1.05	2.40E−34	3.98E−33
ZNF114	1.05	3.01E−18	2.13E−17
FAM35A	1.05	9.15E−46	2.38E−44
SIX2	1.05	0.000341596	0.000705559
ETS1	1.05	3.20E−96	3.24E−94
ERVK13-1	1.05	7.87E−08	2.45E−07
LOC100288123	1.05	0.003598851	0.006403414
SPATA13	1.05	5.28E−19	3.92E−18
PTGER2	1.06	9.55E−10	3.53E−09
METTL12	1.06	1.30E−08	4.37E−08
GNB3	1.06	0.000318505	0.000659914
NOG	1.06	0.001463904	0.002772409
LOC100379224	1.06	1.78E−12	8.34E−12
ZNF514	1.06	2.92E−18	2.07E−17
LOC100506649	1.06	1.27E−46	3.41E−45
CHORDC1	1.07	1.72E−57	6.60E−56
CDKN1A	1.07	7.51E−66	3.68E−64
ARMCX4	1.07	1.38E−14	7.62E−14
NBPF1	1.07	1.78E−24	1.86E−23
TJP2	1.07	8.04E−145	1.75E−142
LOC147804	1.07	1.07E−13	5.51E−13
PRDM13	1.07	2.87E−07	8.40E−07
SON	1.07	2.20E−170	6.31E−168
EPHB2	1.07	6.87E−09	2.37E−08
POM121C	1.08	7.93E−52	2.55E−50
ZNF443	1.08	2.53E−07	7.47E−07
JRK	1.08	7.15E−24	7.18E−23
KBTBD8	1.08	0.000226507	0.000478711
ASB16	1.08	1.81E−06	4.90E−06
FAM86B1	1.08	4.25E−08	1.36E−07
CREB5	1.08	8.91E−16	5.35E−15
VAMP1	1.08	4.97E−14	2.62E−13
USP32P1	1.08	3.37E−24	3.47E−23
IRGQ	1.08	4.72E−37	8.74E−36
RPS26	1.09	8.51E−106	1.02E−103
CLCN4	1.09	2.07E−11	8.92E−11
DPY19L2	1.09	1.60E−06	4.36E−06
TMPPE	1.10	0.000159821	0.000344921
RP9P	1.10	1.53E−14	8.36E−14
ZNF600	1.10	2.10E−16	1.32E−15
C17orf51	1.10	5.46E−47	1.48E−45
ABL2	1.10	6.59E−118	1.02E−115
ZRSR2	1.11	1.52E−05	3.70E−05
ATAD3B	1.11	3.55E−17	2.34E−16
ZBTB26	1.11	5.88E−14	3.08E−13
LOC100527964	1.11	0.000167054	0.00035964
RASSFIO	1.11	0.001652544	0.003100419
WDR52	1.12	0.001376432	0.002616151
ENTPD7	1.12	2.86E−59	1.16E−57
KLHL21	1.12	1.76E−47	4.90E−46
SERPINB7	1.13	5.41E−06	1.39E−05
TFCP2L1	1.13	4.61E−16	2.82E−15
RFTN1	1.13	4.04E−18	2.83E−17
PTHLH	1.13	1.75E−07	5.26E−07
C10rf216	1.13	3.58E−29	4.66E−28
MGC57346	1.13	1.40E−09	5.11E−09
MALAT1	1.14	3.24E−113	4.51E−111
NSUN5P1	1.14	1.10E−06	3.03E−06
C3orf52	1.14	8.59E−13	4.12E−12
MRPS25	1.14	4.61E−67	2.35E−65
C11orf41	1.14	1.89E−08	6.29E−08
EPHA4	1.14	1.27E−05	3.12E−05
LOC283624	1.14	1.05E−22	9.88E−22
FRMD6	1.15	1.79E−118	2.83E−116
XRCC2	1.15	5.36E−36	9.41E−35
ATF5	1.15	7.98E−35	1.34E−33
NOV	1.15	0.000586861	0.00117642
RPL23AP64	1.15	0.000182902	0.00039146
FGF5	1.15	7.83E−05	0.00017528
DNAH17	1.16	5.10E−06	1.31E−05
PPARGC1B	1.16	8.77E−12	3.89E−11
PEA15	1.16	1.76E−127	3.19E−125
MIR22HG	1.16	1.55E−27	1.89E−26
LOC219731	1.16	5.88E−05	0.000133586
SLC7A2	1.16	4.67E−05	0.000107518
ZEB1	1.16	2.44E−37	4.58E−36
MOB3C	1.17	1.91E−22	1.77E−21
SBDSP1	1.17	6.56E−27	7.74E−26
LCAT	1.17	3.08E−08	9.99E−08
HBEGF	1.17	2.63E−45	6.70E−44
MGC70870	1.17	1.69E−109	2.14E−107
CDC42EP2	1.18	6.61E−33	1.03E−31
LOC440300	1.18	1.60E−20	1.31E−19
TMED10P1	1.19	1.35E−14	7.42E−14
B3GALT5	1.19	0.000161499	0.000348482
BMPER	1.19	4.12E−14	2.19E−13
HERC2P7	1.19	1.08E−15	6.44E−15
SEMA3A	1.20	3.81E−10	1.46E−09
HNRNPU-AS1	1.20	1.86E−28	2.35E−27
C20orf118	1.20	1.01E−08	3.43E−08
LOC154761	1.21	1.58E−05	3.84E−05
BTBD6	1.21	3.31E−124	5.74E−122
ALG10	1.21	3.97E−15	2.28E−14
LINC00338	1.22	2.07E−17	1.38E−16
RPL23AP7	1.22	5.87E−08	1.85E−07
CLDN15	1.22	3.94E−17	2.60E−16
TUBA4A	1.22	3.74E−64	1.69E−62
ZNF860	1.22	8.13E−19	5.96E−18
NBPFIO	1.23	1.33E−13	6.78E−13
EFNB2	1.23	5.00E−20	3.98E−19
C15orf52	1.24	3.83E−77	2.56E−75
RRS1	1.24	1.88E−119	3.05E−117
OXTR	1.24	1.09E−16	6.95E−16
CRMP1	1.25	1.15E−06	3.18E−06
ZNF440	1.25	3.58E−18	2.52E−17
EIF4EBP3	1.26	0.000648201	0.001292417
DUSP7	1.26	1.51E−29	1.99E−28
EXOG	1.26	8.37E−22	7.50E−21
MAMLD1	1.27	1.94E−25	2.14E−24
SMPD3	1.27	6.68E−09	2.31E−08
PNN	1.28	1.20E−105	1.42E−103
PMEPA1	1.28	1.35E−87	1.14E−85
SCARF1	1.29	2.58E−10	1.00E−09
LOC100505815	1.29	1.06E−07	3.24E−07
FBXL19-AS1	1.29	1.55E−13	7.86E−13
HIST1H4H	1.29	1.12E−08	3.80E−08
TUBB	1.30	1.63E−74	9.98E−73
LOC100289230	1.30	0.000190102	0.000406114
FAM111B	1.30	2.83E−117	4.27E−115
ZNF33B	1.30	1.31E−57	5.07E−56
ZNF121	1.30	1.57E−28	1.99E−27
ZNF780A	1.30	1.31E−25	1.45E−24
NEFM	1.30	2.06E−10	8.07E−10
DGCR11	1.30	1.08E−13	5.52E−13
ST20	1.31	5.79E−08	1.83E−07
ADAM1	1.32	7.15E−10	2.68E−09
SRSF1	1.32	1.55E−170	4.54E−168
LOC642846	1.33	2.29E−09	8.22E−09
LOC730755	1.33	4.02E−07	1.16E−06
ZNF594	1.33	2.96E−18	2.10E−17
ITGA2	1.33	7.36E−102	8.02E−100
RRN3P3	1.33	4.14E−10	1.58E−09
MXD1	1.34	2.31E−28	2.90E−27
PKI55	1.35	3.94E−10	1.51E−09
LOC100507433	1.35	9.28E−07	2.58E−06
PPAPDC1A	1.35	5.00E−27	5.95E−26
PIGW	1.35	7.76E−78	5.28E−76
NBPF9	1.35	3.18E−46	8.45E−45
ZNF782	1.36	4.73E−11	1.97E−10
RRP7B	1.36	4.18E−08	1.33E−07
MICA	1.36	1.68E−22	1.56E−21
SCARNA12	1.36	4.69E−05	0.000108075
DDX12P	1.36	1.67E−09	6.07E−09
RPSAP9	1.36	5.75E−08	1.81E−07
PLEKHM1	1.37	2.14E−08	7.05E−08
CLDN1	1.37	1.78E−123	2.99E−121
TUBB1	1.37	1.35E−07	4.10E−07
SERHL	1.37	2.41E−09	8.64E−09
YY2	1.38	1.99E−15	1.16E−14
LOC344595	1.38	8.64E−13	4.14E−12
LOC654342	1.39	1.44E−08	4.83E−08
HCN2	1.39	1.10E−05	2.71E−05
TSSK2	1.39	5.96E−06	1.52E−05
SERTAD4	1.40	4.02E−18	2.81E−17
PTGDR2	1.40	1.65E−14	8.98E−14
HTR7P1	1.40	5.99E−16	3.63E−15
C10rf63	1.41	2.97E−32	4.44E−31
OBFC2A	1.41	1.26E−72	7.20E−71
ICOSLG	1.41	3.58E−23	3.46E−22
PPP1R3E	1.42	8.05E−22	7.22E−21
F13A1	1.42	1.56E−21	1.37E−20
WASH1	1.43	1.32E−13	6.69E−13
GNRH1	1.43	7.51E−09	2.59E−08
TLR2	1.44	2.19E−07	6.50E−07
PXDN	1.45	2.57E−18	1.83E−17
LOX	1.45	4.42E−65	2.1 IE−63
EIF3C	1.46	6.70E−21	5.60E−20
EIF3CL	1.46	6.74E−21	5.62E−20
DHRS4L2	1.46	4.24E−11	1.78E−10
CD274	1.46	6.02E−65	2.85E−63
LOC646329	1.46	4.89E−07	1.40E−06
ZNF767	1.46	6.55E−20	5.19E−19
SPNS2	1.46	4.72E−21	3.99E−20
LOC401431	1.47	1.10E−13	5.62E−13
SHISA7	1.47	1.52E−07	4.60E−07
WASH3P	1.47	1.01E−15	6.05E−15
C12orf34	1.47	6.88E−13	3.32E−12
LOC728643	1.47	6.16E−10	2.32E−09
PI4KAP1	1.48	3.1 IE−15	1.80E−14
HSPA8	1.48	0	0
BCL2A1	1.48	1.71E−11	7.41E−11
ADAMTS6	1.49	3.58E−06	9.33E−06
SPIN2B	1.49	1.12E−09	4.12E−09
TUBAIB	1.49	4.03E−278	2.32E−275
TNFAIP3	1.50	3.05E−159	7.86E−157
CCDC39	1.50	3.75E−05	8.72E−05
WASH5P	1.51	3.91E−50	1.19E−48
SPHK1	1.52	4.08E−27	4.87E−26
ZNF417	1.53	1.87E−17	1.25E−16
LOC100289019	1.53	3.60E−10	1.38E−09
TUBA1C	1.54	0	0
BMS1P5	1.54	2.70E−06	7.16E−06
BMS1P1	1.54	2.71E−06	7.17E−06
SHISA9	1.54	1.40E−48	4.04E−47
SOX9	1.54	1.14E−50	3.52E−49
ENC1	1.55	3.65E−104	4.24E−102
PLEKHA8P1	1.55	4.63E−10	1.76E−09
NEAT1	1.55	2.74E−226	1.24E−223
LOC100506123	1.55	6.41E−12	2.88E−11
LOC100506599	1.56	1.30E−08	4.37E−08
FOXO3B	1.56	4.19E−17	2.76E−16
PDIA3P	1.56	1.88E−19	1.44E−18
MMP1	1.56	6.14E−19	4.53E−18
FERMT1	1.57	9.62E−144	2.06E−141
NPTX2	1.57	2.39E−07	7.07E−07
MSTO2P	1.58	3.96E−15	2.27E−14
ZFP112	1.58	2.53E−18	1.80E−17
AFG3L1P	1.59	3.41E−74	2.05E−72
TFRC	1.60	1.56E−210	6.16E−208
SPRN	1.63	3.15E−21	2.70E−20
LOC100133091	1.64	4.17E−08	1.33E−07
GKN2	1.64	1.01E−19	7.88E−19
LOC100272217	1.64	8.20E−08	2.54E−07
LOC100132247	1.66	1.26E−11	5.53E−11
UBC	1.67	0	0
LOC440894	1.69	4.98E−07	1.42E−06
HERC2P2	1.70	1.86E−84	1.51E−82
CBWD2	1.70	2.67E−09	9.56E−09
DQX1	1.71	1.13E−10	4.53E−10
CHRM3	1.72	1.40E−14	7.68E−14
TMEM158	1.72	1.85E−29	2.44E−28
G0S2	1.73	4.26E−70	2.29E−68
LOC100288778	1.77	5.61E−13	2.74E−12
CCZ1	1.81	6.97E−29	9.02E−28
GPR89A	1.81	6.1 IE−21	5.12E−20
DOK3	1.83	1. OOE−10	4.05E−10
C6orf141	1.85	7.97E−58	3.1 IE−56
NAV3	1.85	1.21E−68	6.33E−67
HERC2P9	1.85	9.07E−20	7.09E−19
GOLGA8B	1.86	5.17E−113	7.03E−111
OPHN1	1.93	1.05E−64	4.92E−63
TAGLN	1.94	2.23E−95	2.24E−93
PRG4	1.95	6.72E−29	8.72E−28
NBPF16	1.95	1.02E−114	1.48E−112
AGAP6	1.96	2.09E−25	2.30E−24
PFN1P2	2.05	1.19E−30	1.65E−29
PPP1R11	2.18	2.28E−16	1.42E−15
HMGA2	2.19	7.78E−56	2.85E−54
SRSFIO	2.22	1.64E−22	1.53E−21
GOLGA8A	2.30	1.62E−88	1.41E−86
LOC100216001	2.32	1.88E−13	9.49E−13
NBPF14	2.34	4.08E−85	3.35E−83
LOC284454	2.42	2.30E−69	1.22E−67
ESM1	2.55	6.08E−25	6.53E−24
LOC613037	2.70	2.83E−23	2.74E−22

GO analysis showed Esrrb-dependent DY131 up-regulated genes were important for regulation of transcription, regulation of apoptosis and proliferation, and a majority of down-regulated genes are related to oxidation and reduction, metabolism and translation elongation (Table 4; Additional file 1: Table S1).

**Table 4:** Gene ontology analysis of Esrrb-dependent DY131-altered genes

Term (down-regulated genes)	Count	P value
GO:0042273 ~ ribosomal large subunit biogenesis	4	0.007
GO:0006297 ~ nucleotide-excision repair, DNA gap filling	4	0.032
GO:0006541 ~ glutamine metabolic process	4	0.042
GO:0009083 ~ branched chain family amino acid catabolic process	5	0.001
GO:0042274 ~ ribosomal small subunit biogenesis	5	0.001
GO:0009081 ~ branched chain family amino acid metabolic process	5	0.004
GO:0006904 ~ vesicle docking during exocytosis	5	0.016
GO:0048278 ~ vesicle docking	5	0.021
GO:0006958 ~ complement activation, classical pathway	5	0.031
GO:0022406 ~ membrane docking	5	0.038
GO:0002455 ~ humoral immune response mediated by circulating immunoglobulin	6	0.008
GO:0009060 ~ aerobic respiration	6	0.014
GO:0006635 ~ fatty acid beta-oxidation	7	0.001
GO:0009062 ~ fatty acid catabolic process	7	0.003
GO:0019395 ~ fatty acid oxidation	7	0.005
GO:0034440 ~ lipid oxidation	7	0.005
GO:0033559 ~ unsaturated fatty acid metabolic process	7	0.019
GO:0009064 ~ glutamine family amino acid metabolic process	7	0.020
GO:0019228 ~ regulation of action potential in neuron	7	0.024
GO:0006289 ~ nucleotide-excision repair	7	0.026
GO:0051591 ~ response to cAMP to cAMP	8	0.002
GO:0006800 ~ oxygen and reactive oxygen species metabolic process	8	0.021
GO:0001508 ~ regulation of action potential	8	0.023
GO:0009566 ~ fertilization	8	0.046
GO:0044242 ~ cellular lipid catabolic process	9	0.013
GO:0007160 ~ cell–matrix adhesion	9	0.032
GO:0060627 ~ regulation of vesicle-mediated transport	9	0.046
GO:0009063 ~ cellular amino acid catabolic process	10	0.002
GO:0045333 ~ cellular respiration	10	0.020
GO:0007568 ~ aging	10	0.040
GO:0016485 ~ protein processing	10	0.044
GO:0006364 ~ rRNA processing	11	0.005
GO:0016072 ~ rRNA metabolic process	11	0.007
GO:0006887 ~ exocytosis	11	0.022
GO:0009310 ~ amine catabolic process	12	0.000
GO:0008203 ~ cholesterol metabolic process	12	0.001
GO:0016125 ~ sterol metabolic process	12	0.003
GO:0042391 ~ regulation of membrane potential	12	0.024
GO:0015980 ~ energy derivation by oxidation of organic compounds	12	0.038
GO:0042254 ~ ribosome biogenesis	13	0.005
GO:0016053 ~ organic acid biosynthetic process	14	0.013
GO:0046394 ~ carboxylic acid biosynthetic process	14	0.013
GO:0022613 ~ ribonucleoprotein complex biogenesis	14	0.038
GO:0016042 ~ lipid catabolic process	15	0.013
GO:0006457 ~ protein folding	15	0.016
GO:0034470 ~ ncRNA processing	15	0.025
GO:0022900 ~ electron transport chain	16	0.000
GO:0032940 ~ secretion by cell	16	0.026
GO:0016054 ~ organic acid catabolic process	17	0.000
GO:0046395 ~ carboxylic acid catabolic process	17	0.000
GO:0034660 ~ ncRNA metabolic process	19	0.008
GO:0006631 ~ fatty acid metabolic process	21	0.000
GO:0046903 ~ secretion	22	0.014
GO:0006414 ~ translational elongation	25	0.000
GO:0008610 ~ lipid biosynthetic process	26	0.002
GO:0006091 ~ generation of precursor metabolites and energy	31	0.000
GO:0006412 ~ translation	38	0.000
GO:0016192 ~ vesicle-mediated transport	39	0.003
GO:0055114 ~ oxidation reduction	54	0.000

Term (up-regulated genes)	Count	P value
GO:0002220 ~ innate immune response activating cell surface receptor signaling pathway	2	0.045
GO:0048712 ~ negative regulation of astrocyte differentiation	2	0.045
GO:0000724 ~ double-strand break repair via homologous recombination	3	0.020
GO:0000725 ~ recombinational repair	3	0.020
GO:0045987 ~ positive regulation of smooth muscle contraction	4	0.001
GO:0045933 ~ positive regulation of muscle contraction	4	0.002
GO:0006940 ~ regulation of smooth muscle contraction	4	0.009
GO:0050768 ~ negative regulation of neurogenesis	4	0.015
GO:0010721 ~ negative regulation of cell development	4	0.018
GO:0006937 ~ regulation of muscle contraction	4	0.050
GO:0048704 ~ embryonic skeletal system morphogenesis	5	0.004
GO:0048706 ~ embryonic skeletal system development	5	0.012
GO:0031344 ~ regulation of cell projection organization	5	0.019
GO:0007411 ~ axon guidance	5	0.035
GO:0051258 ~ protein polymerization	6	0.000
GO:0043623 ~ cellular protein complex assembly	6	0.039
GO:0050767 ~ regulation of neurogenesis	6	0.042
GO:0048705 ~ skeletal system morphogenesis	7	0.002
GO:0060284 ~ regulation of cell development	7	0.031
GO:0007018 ~ microtubule-based movement	8	0.000
GO:0051960 ~ regulation of nervous system development	8	0.007
GO:0006916 ~ anti-apoptosis	8	0.010
GO:0007017 ~ microtubule-based process	8	0.027
GO:0001501 ~ skeletal system development	9	0.031
GO:0006917 ~ induction of apoptosis	9	0.031
GO:0012502 ~ induction of programmed cell death	9	0.032
GO:0045596 ~ negative regulation of cell differentiation	10	0.001
GO:0040008 ~ regulation of growth	10	0.017
GO:0043066 ~ negative regulation of apoptosis	10	0.021
GO:0043069 ~ negative regulation of programmed cell death	10	0.023
GO:0060548 ~ negative regulation of cell death	10	0.023
GO:0022403 ~ cell cycle phase	10	0.049
GO:0006928 ~ cell motion	12	0.021
GO:0042127 ~ regulation of cell proliferation	17	0.018
GO:0042981 ~ regulation of apoptosis	18	0.011
GO:0043067 ~ regulation of programmed cell death	18	0.012
GO:0010941 ~ regulation of cell death	18	0.012
GO:0006355 ~ regulation of transcription, DNA-dependent	29	0.047
GO:0051252 ~ regulation of RNA metabolic process	30	0.038
GO:0006350 ~ transcription	43	0.000
GO:0045449 ~ regulation of transcription	48	0.001

## Discussion

Esrrb has gained lots of attention in recent years because of its biological function in stem cells and its ability to reprogram somatic cells to iPSC with *oct4* and *sox2* [6, 13, 17–21]. Several other functions of Esrrb have also been discovered including alteration of energy balance, estrogen receptor and glucocorticoid receptor transcription function modulation, Keap1-Nrf2 signaling inhibition, and tumorigenesis in prostate cancer and endometrial adenocarcinoma [9–12, 22–25]. But transcriptome-wide Esrrb function and Esrrb-regulated genes in cancer cells are not well studied.

Esrrb was reported by Chan et al. as a tumor suppressor in DU145 and LNCaP prostate cancer cells using both in vitro and in vivo models [9]. Expression of Esrrb induced *p21*/*cdkn1a* by directly binding to an ERRE in *p21*/*cdkn1a*’s promoter, arrested cell cycle at S-phase, and significantly inhibited cell growth [9, 26]. Interestingly, we did not find p21/cdkn1a up-regulation after Esrrb expression alone, but after we treated DU145 cells with 3 μM DY131, we observed a significant increase of *p21*/*cdkn1a* mRNA (Table 3; Additional file 2: Figure S1). Scrutinizing the data revealed that Chan’s lab cultured their cells with full serum, while we used charcoal-stripped serum for cell culture and DY131 treatment [9]. This implies that there is a compound or factor that can be removed by charcoal treatment modulated Esrrb’s activity [27, 28].

From the Esrrb-regulated gene list, we found a few target genes that are related to the known function of Esrrb. *Kiaa1199* encoded gene product has been shown to associate with cellular mortality. A *kiaa1199* mutation was reported to relate to nonsyndromic hearing loss. Considering the significant effect of Esrrb mutations on human hearing loss, *kiaa1199* could be a mediator of Esrrb mutant related hearing loss [29–32] [33–36]. Another interesting Esrrb responsive gene is *tagln* (Transgelin). It was inhibited by Esrrb while DY131 treatment relieved the inhibition. *Tagln* was reported to promote DU145 cell migration and invasion, indicating Esrrb can also affect DU145 cell behavior by affecting *tagln* [37].

Judging by the numbers of altered genes induced by Esrrb with or without DY131, and the result that DY131 did not alter any mRNA in the absence of Esrrb, we conclude that DY131 activity is Esrrb-dependent.

## Conclusions

In conclusion, we characterized the transcriptome alteration induced by Esrrb expression as well as Esrrb with its ligand DY131 in prostate cancer cells. We conclude Esrrb-target synthetic ligand requires Esrrb to generate its gene expression modulation effect. Finally, analysis of Esrrb target genes indicates Esrrb may be an important factor in regulating cell proliferation.

## Methods

### Cell culture and reagents

DU145 (ATCC Number: HTB-81) and HEK293 (ATCC number: CRL-1571) cells were obtained from the American Type Culture Collection (ATCC). DU145 cells were cultured in RPMI1640 media (Invitrogen, Grand Island, NY, USA) with 10 % Fetal Bovine Serum (FBS) (GE Healthcare Life Sciences, Logan, UT, USA). HEK293 cells were cultured in Eagle’s Minimal Essential Medium (DMEM) (Invitrogen, Grand Island, NY, USA) with 10 % FBS. 70 % confluent DU145 cells were transfected with either pcDNA3.1-zeo (+)-Esrrb expression vector [4], or control empty vector pcDNA3.1-zeo (+) (Promega, Madison, WI, USA). Empty vector or Esrrb expression vector transfected DU145 cells were maintained in medium containing 150 μg/ml Zeocine (Invitrogen, Grand Island, NY, USA) for 3 weeks for selection. Two biological replicates of DU145 cells transfected with Esrrb were pooled together respectively and were named DU145-Esrrb. Two biological replicates of DU145 cells transfected with control vector were pooled together respectively and were named DU145-pc3.1. Total RNA and protein were collected from cells after they are confluent in 60 mm petri dishes, cultured with phenol-red free RPMI1640 with 10 % Charcoal-stripped FBS [38]. For DY131 (Tocris Bioscience, Bristol, UK) treatment, cells are plated in 60 mm petri dishes until confluent; DU145-pc3.1 and DU145-Esrrb are incubated with 3 μM DY131 diluted in medium with charcoal-stripped FBS for indicated length of time.

### Western-blot

Total protein was isolated from DU145-pc3.1, and DU145-Esrrb cells. 20 μg protein was loaded on 9 % SDS gels. After the proteins were transferred to nitrocellulose membrane, the membrane was blocked and then incubated with 1:2000 diluted monoclonal anti-Esrrb mouse IgG (R&D system, Cat. no: PP-H6705-00) and 1:2000 diluted polyclonal anti-GPADH rabbit IgG (Santa Cruz, Dallas, TX, USA, Cat. no: sc-25777) at 4 degrees overnight. The membrane was then washed and incubated with anti-mouse or anti-rabbit secondary antibody. Chemoluminescence (Promega, Madison, WI, USA) signals were collected using x-ray films (Fisher Scientific, Pittsburg, PA, USA).

### Reverse transcriptase PCR and quantitative PCR

Total RNA was isolated and purified from DU145-pc3.1 and DU145-Esrrb using RNeasy kit (Qiagen, Venlo, Netherlands). 1000 ng of total RNA was used to create cDNA libraries using Superscript III Reverse Transciptase with random primers and oligodT (Invitrogen, Grand Island, NY, USA). Esrrb mRNA concentration was determined using quantitative PCR (qPCR) (iQ SYBR, BioRad, Hercules, CA, USA) on ABI7500 system (Applied Biosystems, Foster City, CA, USA). PCR condition: 95°, 30 s; 60°, 40 s; 72°, 40 s. Each qPCR test was performed three times on each of the two biological replicates. Primer sequences: *zcwpw2* (Genbank: NM_001040432): forward primer: AACAGGGTTGTCTGTGAGACGGA; reverse primer: TGCAGGAGCTTCTGGGCTGC. *hoxb8* (Genbank: NM_024016): forward primer: GATGCGCC CGCAAGCAGC; reverse primer: CCCAGGGCGTGCGATACCTC. *tagln* (Genbank: NM_001001522): forward primer: ATGCCCCGGATGACTTGGCT; reverse primer: GCCATGTCTGGGGAAAGCTCCT. *f13a1* (Genbank: NM_000129): forward primer: TGTTCCGTGAAATCCGGCCC; reverse primer: TGCACGTCCAG CTCGCCATA. *pxdn* (Genbank: NM_012293): forward primer: GCAAGCATTTAA GGGACTTGCCTCT; reverse primer: GCAAAAATAGCCTCTCGAGCTTCGG. *aox1* (Genbank: NM_001159): forward primer: TACGTGAACGGCCGCAAGGT; reverse primer: TGGCTGGGTGATGCCTTATCCT. *bmp4* (Genbank: NM_001202): forward primer: CCACCACGAAGAACA TCTGGAG; reverse primer: GCCCCTTTCCCAATCAGGGC. *tgfβ*: (Genbank: NM_000660) forward primer: AGTGGACATC AACGGGTTCAC; reverse primer: CGCACGCAGCAGTTCTTCTC. *gapdh*: (Genbank: NM_001256799); forward primer: ACCCACTCCTCCACCTTTG; reverse primer: CTCTTGTGCTCTTGCTGGG. *Esrrb*: (Genbank: NM_004452) forward primer: CAAGAAGCTCAAGGTGGAGAAGGAGGAG; reverse primer: CGGTCTGTCC GTTTGTCTGTCTGTAGGT. *Esrrg*: (Genbank: NM_001134285) forward primer: ACCATGAATGGCCATCAGA A; reverse primer: ACCAGCTGAGGGTTCAGGTAT.

### Deep sequencing and differentially expressed genes

2500 ng total RNA from two biological replicates was used to generate cDNA libraries using TruSeq Stranded mRNA Sample Preparation kits (Illumina, San Diego, CA, USA). RNA quality and fragment sizing of cDNA library were determined by the University of Missouri DNA core. Deep sequencing was performed by the MU DNA core using Illumina HiSeq 2000 following the manufacture’s instruction. Briefly, samples (8 total) were pooled into one lane with each sample annealed to a specific indexed adaptor. 50 bp single end reads were generated. For each sample, approximately 18 million reads were generated in.fastq format (NCBI-GEO, accession number: GES71208). The sequencing reads were trimmed and filtered using FASTX-Toolkit (V 0.0.13) (http://hannonlab.cshl.edu/fastx_toolkit), and mapped to genome (UCSC hg18) using TopHat2 [39, 40]. Gene expression values were determined by gene raw read counts using an in-house tool MULTICOM-MAP [41–43]. Raw reads were normalized to each sample’s library size and differentially expressed genes were calculated using R/Bioconductor package edgeR [44]. Specifically, we kept the genes that have at least 1 count-per-million (cpm) in at least 2 samples and computed the effective library sizes. Pairwise gene expression tests were carried out using exact test. Differentially expressed genes were determined by log2 fold change (Log2FC) (Log2FC ≥ 1, or Log2FC ≤ −1), p value (p < 0.05) and false discovery rate (FDR < 0.05) [45].

### Gene set function enrichment

Gene ontology (GO) analysis was performed using DAVID bioinformatics sources 6.7 [46, 47]. Differentially expressed genes from certain pairwise comparisons were uploaded to DAVID server (http://david.abcc.ncifcrf.gov) and GO analysis were performed for biological process (BP). Minimum counts were set as default value (two counts) and maximum EASE score (*p* value) was set to 0.05. Differentially expressed genes pathway enrichment analysis was performed using Kyoto Encyclopedia of Genes and Genomes (KEGG) pathway [48, 49]. Gene expression profiles Spearman ranking correlation analysis was analyzed using R (version 3.0.2). Gene expression heat map and hierarchical clustering were created by R/Bioconductor (version 2.13) package gplot.

### Statistical analysis

qPCR experiments were performed in triplicate on both biological replicates. T test was employed to statistically analyze whether the differences in gene expression is significant (p < 0.05). Statistical significance of gene set overlap (Venn Diagram) are tested according to previous reported method [17].

## Availability of supporting data

The data sets supporting the results of this article are available in the NCBI-GEO repository, accession number: GSE71208, URL: http://www.ncbi.nlm.nih.gov/geo/query/acc.cgi?acc=GSE71208.

## Additional files

10.1186/s12867-015-0049-1 DY131-activated Esrrb regulates p21. **Figure S2.** Full gel images.
10.1186/s12867-015-0049-1 Gene ontology analysis result. **Table** **S2.** Esrrb expression with DY131 treatment (control vs. Esrrb + DY131).

## Abbreviations

Esrrb: estrogen related receptor beta
ERRE: estrogen related receptor response element
SFRE: steroid factor response element
iPSC: induced pluripotent stem cells
OSKM: Oct4, Sox2, Klf4, cMyc
FBS: fetal bovine serum
RT: reverse transcriptase
KEGG: Kyoto encyclopedia of genes and genomes
GO: gene ontology
FC: fold change
Esrrg: estrogen related receptor gamma
