# Supplementary material for: Messenger RNA profile analysis deciphers new Esrrb responsive genes in prostate cancer cells
Source: BMC Mol Biol. 2015 Dec 1;16:21. doi: 10.1186/s12867-015-0049-1 (PMC4667504; doi:10.1186/s12867-015-0049-1)
Supplement: Supplementary file 1 — 10.1186/s12867-015-0049-1 DY131-activated Esrrb regulates p21. Figure S2. Full gel images. [file 12867_2015_49_MOESM1_ESM.pdf]

Supplement Table 1. Gene Ontology analysis results

Esrrb regulated genes gene ontology analysis

Term	Count	PValue
GO:0060284~regulation of cell development	5	0.00637
GO:0006955~immune response	8	1.16E-02
GO:0009611~response to wounding	7	0.01177
GO:0042542~response to hydrogen peroxide	3	0.01748
GO:0050767~regulation of neurogenesis	4	0.02214
GO:0006800~oxygen and reactive oxygen species metabolic process	3	0.02447
GO:0060052~neurofilament cytoskeleton organization	2	0.02862
GO:0000302~response to reactive oxygen species	3	0.03016
GO:0051960~regulation of nervous system development	4	0.03218
GO:0031960~response to corticosteroid stimulus	3	0.03792
GO:0010035~response to inorganic substance	4	0.03796
GO:0045661~regulation of myoblast differentiation	2	0.03914
GO:0048667~cell morphogenesis involved in neuron differentiation	4	0.03984

DY131 down regulated genes gene ontology analysis (DY131+Esrrb vs. Esrrb)

Term	Count	PValue
GO:0006414~translational elongation	25	2.34E-12
GO:0006412~translation	38	4.24E-08
GO:0055114~oxidation reduction	54	1.19E-06
GO:0016054~organic acid catabolic process	17	1.36E-05
GO:0046395~carboxylic acid catabolic process	17	1.36E-05
GO:0006091~generation of precursor metabolites and energy	31	1.85E-05
GO:0022900~electron transport chain	16	7.46E-05
GO:0006631~fatty acid metabolic process	21	2.26E-04
GO:0009310~amine catabolic process	12	3.64E-04
GO:0009083~branched chain family amino acid catabolic process	5	7.75E-04
GO:0042274~ribosomal small subunit biogenesis	5	7.75E-04
GO:0006635~fatty acid beta-oxidation	7	8.71E-04
GO:0008203~cholesterol metabolic process	12	0.00149
GO:0051591~response to cAMP to cAMP	8	0.00158
GO:0009063~cellular amino acid catabolic process	10	0.00194
GO:0008610~lipid biosynthetic process	26	0.00218
GO:0016125~sterol metabolic process	12	0.00315
GO:0009062~fatty acid catabolic process	7	0.0034
GO:0016192~vesicle-mediated transport	39	0.00342
GO:0009081~branched chain family amino acid metabolic process	5	0.00362
GO:0006364~rRNA processing	11	0.00484
GO:0042254~ribosome biogenesis	13	0.00484
GO:0019395~fatty acid oxidation	7	0.00512
GO:0034440~lipid oxidation	7	0.00512
GO:0016072~rRNA metabolic process	11	0.00654
GO:0042273~ribosomal large subunit biogenesis	4	0.00691
GO:0034660~ncRNA metabolic process	19	0.00767
GO:0002455~humoral immune response mediated by circulating immunoglobulin	6	0.0085
GO:0016053~organic acid biosynthetic process	14	0.01272
GO:0046394~carboxylic acid biosynthetic process	14	0.01272
GO:0016042~lipid catabolic process	15	0.01334
GO:0044242~cellular lipid catabolic process	9	0.01346
GO:0046903~secretion	22	0.0141
GO:0009060~aerobic respiration	6	0.01417
GO:0006457~protein folding	15	0.01602
GO:0006904~vesicle docking during exocytosis	5	0.01625
GO:0033559~unsaturated fatty acid metabolic process	7	0.01861
GO:0045333~cellular respiration	10	0.01954

GO:0009064~glutamine family amino acid metabolic process	7	0.02033
GO:0006800~oxygen and reactive oxygen species metabolic process	8	0.02096
GO:0048278~vesicle docking	5	0.02143
GO:0006887~exocytosis	11	0.02156
GO:0001508~regulation of action potential	8	0.02256
GO:0019228~regulation of action potential in neuron	7	0.02406
GO:0042391~regulation of membrane potential	12	0.02409
GO:0034470~ncRNA processing	15	0.02451
GO:0006289~nucleotide-excision repair	7	0.0261
GO:0032940~secretion by cell	16	0.02639
GO:0006958~complement activation, classical pathway	5	0.03088
GO:0006297~nucleotide-excision repair, DNA gap filling	4	0.03155
GO:0007160~cell-matrix adhesion	9	0.03171
GO:0022613~ribonucleoprotein complex biogenesis	14	0.03786
GO:0015980~energy derivation by oxidation of organic compounds	12	0.03803
GO:0022406~membrane docking	5	0.03835
GO:0007568~aging	10	0.03978
GO:0006541~glutamine metabolic process	4	0.0423
GO:0016485~protein processing	10	0.04382
GO:0009566~fertilization	8	0.0459
GO:0060627~regulation of vesicle-mediated transport	9	0.04648

DY131 up regulated genes gene ontology analysis (DY131+Esrrb vs. Esrrb)

Term	Count	PValue
GO:0006350~transcription	43	1.47E-04
GO:0051258~protein polymerization	6	2.35E-04
GO:0007018~microtubule-based movement	8	3.17E-04
GO:0045449~regulation of transcription	48	5.77E-04
GO:0045596~negative regulation of cell differentiation	10	8.58E-04
GO:0045987~positive regulation of smooth muscle contraction	4	0.00106
GO:0045933~positive regulation of muscle contraction	4	0.00169
GO:0048705~skeletal system morphogenesis	7	0.00181
GO:0048704~embryonic skeletal system morphogenesis	5	0.00409
GO:0051960~regulation of nervous system development	8	0.00667
GO:0006940~regulation of smooth muscle contraction	4	0.00928
GO:0006916~anti-apoptosis	8	0.00959
GO:0042981~regulation of apoptosis	18	0.01067
GO:0043067~regulation of programmed cell death	18	0.01169
GO:0048706~embryonic skeletal system development	5	0.01175
GO:0010941~regulation of cell death	18	0.01209
GO:0050768~negative regulation of neurogenesis	4	0.01473
GO:0040008~regulation of growth	10	0.01673
GO:0010721~negative regulation of cell development	4	0.01751
GO:0042127~regulation of cell proliferation	17	0.01838
GO:0031344~regulation of cell projection organization	5	0.01909
GO:0000724~double-strand break repair via homologous recombination	3	0.01963
GO:0000725~recombinational repair	3	0.01963
GO:0043066~negative regulation of apoptosis	10	0.02078
GO:0006928~cell motion	12	0.02081
GO:0043069~negative regulation of programmed cell death	10	0.02251
GO:0060548~negative regulation of cell death	10	0.02287
GO:0007017~microtubule-based process	8	0.02656
GO:0001501~skeletal system development	9	0.03058
GO:0060284~regulation of cell development	7	0.03068
GO:0006917~induction of apoptosis	9	0.03108
GO:0012502~induction of programmed cell death	9	0.03157
GO:0007411~axon guidance	5	0.03452

GO:0051252~regulation of RNA metabolic process	30	0.0378
GO:0043623~cellular protein complex assembly	6	0.03876
GO:0050767~regulation of neurogenesis	6	0.04217
GO:0002220~innate immune response activating cell surface receptor signaling pathwa[illegible]	2	0.04505
GO:0048712~negative regulation of astrocyte differentiation	2	0.04505
GO:0006355~regulation of transcription, DNA-dependent	29	0.04716
GO:0022403~cell cycle phase	10	0.04883
GO:0006937~regulation of muscle contraction	4	0.0496

Supplement Table 2. Esrrb expression with DY131 treatment (control vs. Esrrb+DY131)

name	logFC	PValue	FDR
MTRNR2L8	-6.70	0	0
MTRNR2L10	-5.18	1.63E-228	7.61E-226
RPS29	-4.90	0	0
SEC61G	-4.58	4.99E-151	1.29E-148
RPL36A	-4.41	4.68E-247	2.57E-244
AOX1	-3.97	2.45E-183	7.93E-181
TMEM212	-3.69	1.35E-54	5.49E-53
RPL12	-3.67	0	0
NEDD8-MDP1	-3.34	1.33E-91	1.35E-89
ESRG	-3.29	4.00E-101	4.55E-99
SLIRP	-3.29	2.29E-123	3.86E-121
WISP2	-3.27	1.57E-63	7.87E-62
FOS	-3.16	2.52E-39	5.97E-38
ASS1	-3.15	1.24E-34	2.35E-33
LGALS3	-3.12	4.47E-110	6.14E-108
MTRNR2L1	-3.09	0	0
MUC1	-2.92	6.80E-166	2.05E-163
OASL	-2.87	7.73E-49	2.67E-47
RPL31	-2.84	0	0
SEPP1	-2.82	9.64E-25	1.10E-23
SYTL1	-2.81	4.65E-25	5.44E-24
EGR1	-2.75	0	0
ACP1	-2.74	3.90E-32	6.51E-31
RARRES3	-2.74	5.90E-23	6.11E-22
NAPSA	-2.71	2.89E-16	1.96E-15
TNNT1	-2.64	1.29E-47	4.20E-46
DDX60	-2.63	1.37E-23	1.46E-22
USMG5	-2.60	7.74E-85	6.44E-83
CYBA	-2.59	3.82E-20	3.36E-19
PSMA1	-2.56	1.06E-92	1.08E-90
RPL13AP5	-2.55	0	0
STAT4	-2.52	1.15E-23	1.24E-22
CCDC152	-2.52	6.81E-19	5.54E-18
MKNK2	-2.52	7.30E-110	9.93E-108
NDUFA11	-2.51	2.10E-105	2.60E-103
CYP4F11	-2.51	3.17E-24	3.54E-23
RPL9	-2.50	0	0

DHRS3	-2.50	3.59E-48	1.19E-46
CRIP1	-2.48	2.51E-144	6.00E-142
RPL18	-2.47	0	0
SCFD1	-2.46	4.15E-259	2.62E-256
KCNK15	-2.45	2.14E-45	6.44E-44
PIP5KL1	-2.45	4.97E-23	5.17E-22
MRPL4	-2.44	8.20E-73	5.37E-71
RPS15A	-2.44	0	0
TCTEX1D2	-2.41	1.13E-18	9.06E-18
MXD3	-2.37	1.94E-38	4.36E-37
ABCD3	-2.37	1.41E-18	1.12E-17
PDCD4	-2.37	6.22E-111	8.84E-109
SEMA6B	-2.36	7.94E-52	3.01E-50
PDE9A	-2.31	3.30E-54	1.32E-52
NDUFB2	-2.29	6.44E-70	3.93E-68
CLIC3	-2.26	1.48E-15	9.48E-15
PADI2	-2.25	1.86E-45	5.65E-44
NPTX1	-2.25	3.90E-41	9.86E-40
RPL34	-2.24	1.27E-252	7.68E-250
ANXA1	-2.22	0	0
BCKDHB	-2.22	1.77E-30	2.73E-29
NEFL	-2.20	5.41E-18	4.15E-17
PIR-FIGF	-2.19	3.34E-33	5.89E-32
IGFBP3	-2.18	1.56E-176	4.80E-174
RPL38	-2.18	5.66E-242	2.98E-239
TYMP	-2.18	1.30E-36	2.67E-35
COX17	-2.16	1.46E-21	1.38E-20
RARS2	-2.15	8.08E-42	2.08E-40
SNRPF	-2.14	2.26E-53	8.83E-52
SUCLG2	-2.14	1.12E-94	1.16E-92
CDK7	-2.14	2.94E-54	1.18E-52
GBP2	-2.14	2.98E-21	2.77E-20
FRA10AC1	-2.11	9.94E-45	2.88E-43
CNTN1	-2.11	1.81E-14	1.06E-13
RPL11	-2.11	0	0
RPS12	-2.11	2.46E-241	1.24E-238
APLP1	-2.10	2.08E-25	2.48E-24
CLDN4	-2.09	8.45E-44	2.36E-42
MACROD1	-2.09	1.08E-13	5.94E-13
LOC728730	-2.08	5.87E-15	3.57E-14
CA11	-2.07	9.63E-19	7.73E-18
PSME1	-2.07	3.05E-95	3.22E-93

LOC1001347[illegible]	-2.06	2.83E-17	2.06E-16
LOC728190	-2.05	2.66E-12	1.31E-11
NOTCH3	-2.05	5.40E-12	2.59E-11
CBLC	-2.04	2.08E-22	2.06E-21
ELMO3	-2.01	7.62E-41	1.91E-39
C17orf61	-2.01	8.24E-72	5.29E-70
NFASC	-2.00	6.61E-10	2.63E-09
TM2D1	-2.00	1.72E-26	2.16E-25
RAB26	-2.00	1.49E-13	8.17E-13
IL17RC	-1.98	8.36E-23	8.54E-22
ELF3	-1.98	2.79E-45	8.33E-44
SPAG4	-1.98	7.45E-24	8.11E-23
PCCA	-1.97	1.27E-22	1.28E-21
AKR1C3	-1.96	3.89E-11	1.72E-10
STX8	-1.96	3.59E-26	4.46E-25
IL11RA	-1.96	9.97E-16	6.49E-15
IL6	-1.96	1.99E-19	1.68E-18
TBC1D17	-1.95	9.33E-26	1.13E-24
ADAM22	-1.94	2.64E-18	2.07E-17
IFI6	-1.94	3.44E-28	4.80E-27
RPL14	-1.94	1.13E-259	7.54E-257
CDK5	-1.94	4.08E-22	3.97E-21
IL20RB	-1.94	1.41E-28	2.00E-27
MT1X	-1.93	8.57E-28	1.17E-26
CEBPD	-1.93	3.24E-78	2.37E-76
HHIPL2	-1.93	5.46E-12	2.61E-11
IFI44	-1.93	2.77E-11	1.25E-10
GHDC	-1.92	5.83E-27	7.56E-26
MTRNR2L2	-1.91	1.06E-217	4.80E-215
APOL1	-1.91	3.09E-15	1.92E-14
DYNC2LI1	-1.90	1.25E-16	8.75E-16
MX1	-1.90	7.51E-34	1.37E-32
SLC6A3	-1.90	3.21E-09	1.20E-08
LOC1005071[illegible]	-1.89	4.75E-22	4.60E-21
EIF3K	-1.89	3.66E-145	9.06E-143
C4orf48	-1.89	1.95E-11	8.92E-11
QARS	-1.89	2.45E-110	3.44E-108
RPA3	-1.88	3.59E-54	1.43E-52
BIK	-1.88	9.61E-13	4.92E-12
RHOV	-1.87	3.72E-43	1.01E-41
ALDH3B1	-1.86	7.57E-79	5.63E-77
TMEM120A	-1.85	1.28E-32	2.19E-31

GBP1	-1.84	7.29E-10	2.89E-09
RPL37	-1.84	0	0
NFE2	-1.84	1.81E-07	5.68E-07
ANO9	-1.84	1.59E-17	1.18E-16
LOC644961	-1.83	4.61E-12	2.22E-11
POLE2	-1.83	5.94E-61	2.80E-59
IFIT3	-1.82	1.55E-46	4.85E-45
GSTM1	-1.82	3.32E-22	3.25E-21
PTH1R	-1.82	1.19E-07	3.82E-07
TRPT1	-1.82	3.02E-32	5.06E-31
TENC1	-1.81	2.82E-12	1.39E-11
KISS1R	-1.80	3.81E-08	1.27E-07
RTN2	-1.80	1.68E-08	5.82E-08
ARRDC3	-1.80	1.53E-124	2.65E-122
OSBPL5	-1.80	8.00E-10	3.16E-09
NSMCE4A	-1.80	1.61E-29	2.38E-28
ACBD4	-1.79	2.61E-28	3.64E-27
VWA5A	-1.79	8.09E-14	4.52E-13
NAPRT1	-1.79	2.43E-80	1.89E-78
TRIM9	-1.79	3.95E-11	1.75E-10
PACSIN1	-1.78	4.22E-09	1.55E-08
NOXA1	-1.78	1.72E-09	6.58E-09
PC	-1.77	1.28E-23	1.37E-22
GTF2H2D	-1.77	4.24E-11	1.87E-10
C11orf80	-1.76	1.57E-15	1.00E-14
IFT52	-1.75	9.65E-37	1.99E-35
PPIH	-1.75	1.22E-17	9.14E-17
DEPTOR	-1.74	2.24E-06	6.26E-06
GLTSCR2	-1.73	2.43E-62	1.18E-60
RUVBL2	-1.73	1.33E-89	1.28E-87
GNB2L1	-1.73	7.84E-302	5.83E-299
ST14	-1.72	4.79E-25	5.59E-24
ETFA	-1.72	6.33E-115	9.53E-113
JPX	-1.71	1.97E-08	6.75E-08
EIF3E	-1.71	5.34E-216	2.33E-213
ANXA6	-1.71	4.65E-86	4.08E-84
SIDT2	-1.70	4.79E-33	8.35E-32
RPLP2	-1.70	2.32E-212	9.48E-210
PDCD5	-1.70	7.01E-43	1.88E-41
WIPI1	-1.70	9.08E-14	5.05E-13
THYN1	-1.70	2.79E-35	5.40E-34
LRSAM1	-1.70	9.95E-34	1.80E-32

PSMA3	-1.70	4.02E-90	3.91E-88
CHCHD6	-1.70	9.67E-15	5.79E-14
STXBP2	-1.70	1.12E-69	6.76E-68
HMGN5	-1.69	1.16E-11	5.38E-11
SYT12	-1.69	7.75E-37	1.61E-35
SNURF	-1.68	4.75E-85	4.03E-83
SNRPN	-1.68	5.47E-85	4.61E-83
AUH	-1.67	1.10E-13	6.09E-13
ATP5D	-1.67	1.10E-39	2.66E-38
LHPP	-1.67	4.22E-09	1.55E-08
DNAJC17	-1.66	1.19E-11	5.51E-11
ACSF2	-1.66	3.34E-09	1.24E-08
ABHD14A-ACY	-1.66	6.59E-32	1.09E-30
RPL35A	-1.66	6.73E-189	2.24E-186
RNF128	-1.65	1.54E-12	7.74E-12
IGFBP6	-1.65	3.28E-53	1.28E-51
IQGAP2	-1.65	4.35E-13	2.28E-12
STX10	-1.64	3.94E-23	4.12E-22
STK32A	-1.64	1.37E-07	4.37E-07
GRAMD2	-1.64	1.84E-07	5.78E-07
FADS3	-1.64	1.02E-64	5.34E-63
TNFRSF9	-1.64	1.10E-08	3.88E-08
GARNL3	-1.64	3.70E-09	1.37E-08
PTPRU	-1.64	3.80E-09	1.40E-08
GRB7	-1.64	8.50E-21	7.73E-20
SDHB	-1.64	4.06E-50	1.46E-48
DLST	-1.63	2.42E-20	2.15E-19
HEXB	-1.63	9.82E-131	1.91E-128
NDUFA1	-1.63	3.58E-51	1.32E-49
WDR54	-1.63	2.72E-33	4.81E-32
FRG1	-1.63	2.00E-09	7.61E-09
NPM1	-1.63	3.87E-202	1.40E-199
PMF1-BGLAP	-1.62	1.86E-43	5.12E-42
C1S	-1.62	6.48E-14	3.66E-13
SEMA3B	-1.62	5.38E-12	2.58E-11
ENDOV	-1.62	2.82E-10	1.16E-09
S100A4	-1.62	1.73E-09	6.62E-09
ADCK4	-1.61	2.48E-24	2.79E-23
IFT27	-1.61	9.43E-19	7.58E-18
C14orf132	-1.61	1.36E-07	4.34E-07
PION	-1.61	9.47E-16	6.18E-15
CHEK2	-1.61	1.39E-36	2.85E-35

SYTL2	-1.61	2.91E-38	6.50E-37
SPC24	-1.61	2.57E-10	1.06E-09
CYP24A1	-1.61	1.18E-27	1.60E-26
USP4	-1.60	6.45E-14	3.65E-13
SUPT3H	-1.60	1.25E-12	6.34E-12
PLA2G6	-1.60	1.31E-11	6.05E-11
C10orf10	-1.60	7.00E-19	5.68E-18
KCNAB2	-1.60	9.07E-27	1.16E-25
TM7SF2	-1.60	1.07E-39	2.60E-38
PNPLA6	-1.59	7.15E-35	1.36E-33
NUCB2	-1.59	9.10E-39	2.09E-37
UROS	-1.59	1.38E-32	2.36E-31
PTP4A3	-1.59	8.93E-12	4.18E-11
CCDC104	-1.59	6.30E-29	9.07E-28
ABCG2	-1.59	8.30E-06	2.16E-05
LOC1002875[illegible]	-1.58	8.67E-07	2.53E-06
LLGL2	-1.58	1.65E-14	9.76E-14
C18orf8	-1.58	1.39E-48	4.75E-47
C9orf84	-1.58	4.70E-48	1.55E-46
ATP6AP1L	-1.57	6.24E-06	1.65E-05
ARPC4-TTLL[illegible]	-1.57	9.77E-27	1.25E-25
SLC38A6	-1.57	5.09E-10	2.05E-09
TMEM110-MU[illegible]	-1.56	1.22E-06	3.48E-06
EFEMP2	-1.56	8.52E-06	2.21E-05
IFIT1	-1.56	5.55E-31	8.74E-30
ALDH4A1	-1.56	5.39E-18	4.15E-17
LOC643406	-1.56	8.39E-15	5.06E-14
ZCRB1	-1.56	3.04E-90	2.98E-88
EPHX2	-1.56	1.83E-14	1.08E-13
DPYSL5	-1.55	8.07E-07	2.36E-06
ZC3H12D	-1.55	1.65E-05	4.13E-05
HOOK2	-1.55	1.75E-27	2.35E-26
ACSS2	-1.55	2.10E-85	1.80E-83
YPEL3	-1.55	4.04E-15	2.49E-14
LGALS3BP	-1.54	2.37E-38	5.32E-37
ASNS	-1.54	1.41E-100	1.59E-98
DTX4	-1.54	1.50E-12	7.56E-12
C15orf48	-1.53	1.99E-56	8.50E-55
C1orf172	-1.53	1.21E-09	4.70E-09
NDUFC1	-1.53	2.54E-39	6.01E-38
C1R	-1.53	2.37E-15	1.49E-14
CACNA2D2	-1.53	3.43E-07	1.05E-06

TCEA2	-1.53	1.84E-31	2.98E-30
WDR83	-1.53	2.01E-21	1.88E-20
C10orf55	-1.53	1.04E-78	7.64E-77
PHGDH	-1.53	1.07E-153	2.81E-151
THOC6	-1.52	8.01E-30	1.20E-28
ANXA4	-1.52	2.03E-103	2.36E-101
C10orf54	-1.52	4.76E-79	3.60E-77
MGST2	-1.52	1.02E-10	4.36E-10
COX7B	-1.51	2.01E-66	1.13E-64
ACY1	-1.51	1.69E-24	1.92E-23
PBXIP1	-1.51	7.39E-67	4.19E-65
NKD2	-1.51	5.27E-12	2.53E-11
PRKCZ	-1.51	8.08E-19	6.53E-18
AASS	-1.51	6.91E-07	2.04E-06
ATPAF2	-1.51	2.05E-07	6.40E-07
GTF2H2C	-1.51	4.52E-08	1.50E-07
RPL37A	-1.51	1.19E-150	3.00E-148
XRCC1	-1.50	9.28E-32	1.52E-30
FBXO16	-1.49	8.35E-06	2.17E-05
GLB1L	-1.49	6.83E-11	2.96E-10
P2RY6	-1.49	2.68E-16	1.83E-15
TMEM45A	-1.49	4.40E-08	1.46E-07
HSPB8	-1.48	2.04E-07	6.39E-07
C19orf79	-1.48	2.88E-15	1.80E-14
RNASEH2B	-1.48	3.02E-21	2.80E-20
C11orf10	-1.48	1.04E-38	2.38E-37
ALDH6A1	-1.48	3.15E-15	1.96E-14
HSD17B4	-1.48	1.19E-90	1.17E-88
FUZ	-1.48	5.40E-10	2.17E-09
COG6	-1.47	8.35E-23	8.54E-22
CCDC125	-1.47	5.42E-15	3.31E-14
PLK2	-1.47	2.50E-105	3.07E-103
UNC93B1	-1.47	3.53E-24	3.94E-23
SLC22A18	-1.46	7.43E-12	3.51E-11
C10orf11	-1.46	6.12E-06	1.62E-05
GSTA4	-1.46	8.75E-18	6.62E-17
GPR108	-1.46	3.04E-38	6.78E-37
NIT2	-1.46	4.87E-27	6.34E-26
ZNF826P	-1.46	7.18E-05	0.00016623
IFI35	-1.46	6.91E-19	5.61E-18
LMBRD1	-1.46	4.67E-26	5.76E-25
RAP1GAP	-1.46	2.08E-11	9.46E-11

IFIT2	-1.46	1.02E-26	1.30E-25
MIR497HG	-1.46	3.53E-14	2.04E-13
PRSS22	-1.46	6.14E-06	1.63E-05
TXNIP	-1.46	2.64E-20	2.33E-19
TCP11L2	-1.46	5.12E-09	1.87E-08
FBXW9	-1.46	1.19E-07	3.80E-07
SRI	-1.45	1.84E-64	9.48E-63
SLC25A5-AS1	-1.45	1.05E-26	1.34E-25
PTGES	-1.45	9.83E-36	1.95E-34
TCTN1	-1.45	1.57E-14	9.33E-14
JMJD8	-1.45	2.75E-26	3.43E-25
PARL	-1.45	1.39E-59	6.36E-58
MEF2BNB-MEI	-1.45	2.73E-07	8.43E-07
TEX9	-1.45	3.68E-06	1.00E-05
PDLIM2	-1.44	1.06E-47	3.44E-46
CBS	-1.44	3.99E-79	3.04E-77
PRKCSH	-1.44	1.32E-104	1.56E-102
LSS	-1.44	3.12E-44	8.93E-43
ZBTB8OS	-1.44	2.39E-21	2.23E-20
ATP5E	-1.44	9.00E-89	8.43E-87
ALDH1L2	-1.44	2.64E-40	6.48E-39
HES1	-1.43	4.38E-15	2.70E-14
NAE1	-1.43	1.29E-69	7.78E-68
CLASRP	-1.43	6.19E-21	5.65E-20
TRAPPC9	-1.43	4.53E-20	3.97E-19
PIR	-1.43	2.35E-31	3.76E-30
IMMP1L	-1.43	5.50E-07	1.64E-06
PDIA5	-1.43	1.73E-17	1.28E-16
TECR	-1.42	1.37E-66	7.72E-65
CRYZL1	-1.42	8.85E-29	1.27E-27
KIAA1456	-1.42	1.30E-08	4.55E-08
HDAC5	-1.42	2.65E-29	3.90E-28
WDR27	-1.42	1.89E-10	7.88E-10
RNF181	-1.42	5.03E-38	1.11E-36
WBSCR22	-1.41	2.55E-57	1.10E-55
PEX7	-1.41	1.95E-11	8.90E-11
LINC00263	-1.41	1.36E-17	1.01E-16
TCIRG1	-1.41	3.80E-34	7.05E-33
ADA	-1.41	1.58E-16	1.09E-15
TBCE	-1.41	6.13E-34	1.13E-32
HSF4	-1.41	2.23E-09	8.45E-09
UPK3B	-1.41	1.32E-12	6.71E-12

PRR5-ARHGAI	-1.41	8.32E-10	3.28E-09
CYS1	-1.41	1.06E-05	2.72E-05
ITFG1	-1.41	1.35E-66	7.60E-65
C6orf70	-1.41	2.78E-21	2.59E-20
FRG1B	-1.41	7.55E-13	3.90E-12
ILDR2	-1.41	4.97E-06	1.33E-05
PTPN6	-1.41	7.83E-30	1.18E-28
CTGF	-1.40	9.60E-43	2.56E-41
ANK2	-1.40	3.65E-21	3.36E-20
EXOSC9	-1.40	3.37E-45	9.98E-44
FBXL16	-1.40	2.20E-11	1.00E-10
NCOA7	-1.40	6.00E-60	2.76E-58
IER5L	-1.40	2.18E-33	3.90E-32
CCDC88B	-1.40	3.54E-16	2.39E-15
MT1F	-1.40	2.35E-05	5.78E-05
PXK	-1.40	2.88E-16	1.95E-15
TMEM205	-1.40	1.68E-37	3.61E-36
LOC1005067	-1.40	1.85E-07	5.80E-07
KCNMB4	-1.40	7.48E-05	0.00017269
JUP	-1.40	5.40E-79	4.06E-77
CERCAM	-1.40	2.69E-69	1.59E-67
CAMK1	-1.40	9.01E-21	8.17E-20
ADAMTS13	-1.40	1.00E-12	5.13E-12
LTBP3	-1.39	2.42E-45	7.26E-44
DMPK	-1.39	4.96E-19	4.08E-18
CXCR4	-1.39	1.10E-06	3.18E-06
PLD1	-1.39	9.93E-12	4.64E-11
UNC5CL	-1.39	1.86E-08	6.40E-08
SAMD9	-1.39	2.67E-21	2.49E-20
LOC1005056	-1.39	4.24E-12	2.05E-11
CRIP2	-1.39	4.39E-12	2.12E-11
PLEKHA6	-1.39	1.29E-10	5.47E-10
CEP70	-1.39	2.84E-46	8.80E-45
FDPS	-1.38	1.28E-127	2.31E-125
RPS24	-1.38	3.17E-164	9.12E-162
COX7C	-1.38	3.83E-103	4.40E-101
IFT140	-1.38	2.38E-15	1.50E-14
GFM2	-1.38	6.38E-89	6.02E-87
ENPEP	-1.38	4.54E-10	1.84E-09
FRMD4B	-1.38	4.75E-06	1.28E-05
RPS7	-1.37	7.04E-133	1.46E-130
FER1L4	-1.37	1.01E-25	1.22E-24

COX5A	-1.37	3.01E-77	2.15E-75
ARHGAP8	-1.37	3.31E-10	1.35E-09
TSNAX-DISC1	-1.37	6.26E-21	5.71E-20
CXCL16	-1.36	1.02E-16	7.19E-16
LMTK3	-1.35	7.08E-14	3.99E-13
TLE2	-1.35	5.20E-28	7.17E-27
TRANK1	-1.35	6.84E-07	2.02E-06
LAMA5	-1.35	3.83E-75	2.63E-73
ERAP2	-1.35	5.35E-71	3.38E-69
ACADS	-1.35	9.69E-16	6.32E-15
GMDS	-1.34	2.63E-20	2.33E-19
GDPD1	-1.34	2.66E-05	6.48E-05
DYX1C1	-1.34	4.04E-07	1.22E-06
NDUFA2	-1.34	1.99E-29	2.92E-28
PARP10	-1.34	1.28E-25	1.54E-24
SNX14	-1.34	1.09E-64	5.66E-63
PRELID2	-1.34	1.73E-07	5.46E-07
PCCB	-1.34	1.89E-63	9.40E-62
FAM162A	-1.34	1.23E-42	3.25E-41
HSCB	-1.33	3.77E-13	1.99E-12
PXDN	-1.33	3.95E-27	5.18E-26
PLCD1	-1.33	1.26E-10	5.34E-10
C11orf67	-1.33	1.20E-06	3.44E-06
TBCA	-1.33	7.32E-65	3.89E-63
IFT43	-1.33	2.70E-10	1.11E-09
ALG5	-1.32	2.16E-16	1.49E-15
GALE	-1.32	2.04E-46	6.34E-45
GRAMD1A	-1.32	2.69E-55	1.12E-53
RNASE4	-1.32	4.65E-33	8.12E-32
C11orf54	-1.32	4.59E-22	4.46E-21
TTC39A	-1.32	3.31E-09	1.23E-08
ESD	-1.32	1.72E-46	5.36E-45
LAMP3	-1.32	3.47E-09	1.29E-08
CRELD2	-1.32	1.60E-17	1.18E-16
MME	-1.32	2.02E-08	6.94E-08
BDH2	-1.32	1.51E-14	8.99E-14
IFITM10	-1.32	1.37E-09	5.29E-09
SUGT1	-1.32	6.07E-34	1.12E-32
MRPL13	-1.32	4.82E-35	9.23E-34
GUK1	-1.32	1.17E-70	7.34E-69
BCKDHA	-1.32	8.60E-55	3.51E-53
SREBF1	-1.32	3.57E-36	7.19E-35

C8orf45	-1.31	3.25E-05	7.84E-05
LEPR	-1.31	9.92E-18	7.47E-17
NDUFA13	-1.31	1.89E-44	5.44E-43
SNX2	-1.31	1.24E-49	4.40E-48
RABGGTA	-1.31	2.89E-12	1.42E-11
IFI27L1	-1.31	2.35E-17	1.72E-16
ULK4	-1.31	0.00017056	0.00037618
CCNA1	-1.31	4.56E-16	3.05E-15
PKN3	-1.30	1.51E-12	7.61E-12
RPS19	-1.30	5.44E-196	1.86E-193
CERS4	-1.30	8.89E-12	4.17E-11
RPS25	-1.30	3.92E-128	7.19E-126
TMEM8B	-1.30	1.12E-18	8.96E-18
GNG7	-1.30	3.91E-06	1.06E-05
STX4	-1.30	6.48E-26	7.93E-25
C19orf71	-1.30	3.01E-09	1.13E-08
BRSK1	-1.30	4.84E-06	1.30E-05
OMA1	-1.30	8.28E-25	9.51E-24
P2RX4	-1.30	7.31E-18	5.55E-17
FAM175A	-1.30	5.48E-05	0.00012876
RPS8	-1.30	2.66E-177	8.41E-175
FAH	-1.30	3.31E-14	1.92E-13
FAM125A	-1.29	1.40E-18	1.12E-17
GAL3ST1	-1.29	6.02E-05	0.00014058
GAPDHS	-1.29	0.00041867	0.00087195
CRYL1	-1.29	9.56E-11	4.09E-10
ANKRD13D	-1.29	2.25E-22	2.22E-21
FBXO2	-1.29	2.51E-18	1.98E-17
DECR1	-1.29	7.24E-28	9.92E-27
BCL3	-1.29	1.12E-39	2.71E-38
PCYOX1L	-1.29	9.00E-30	1.35E-28
BBS5	-1.29	1.06E-27	1.44E-26
NPM3	-1.28	1.61E-27	2.17E-26
ALKBH7	-1.28	1.22E-22	1.22E-21
FLJ22184	-1.28	1.99E-12	9.91E-12
UBXN11	-1.28	1.98E-05	4.89E-05
DUT	-1.28	8.47E-64	4.27E-62
PYROXD2	-1.28	2.04E-08	7.00E-08
PTPRH	-1.28	1.72E-14	1.02E-13
MAP2K5	-1.28	2.27E-09	8.58E-09
SEMA6C	-1.27	2.61E-07	8.06E-07
FGGY	-1.27	1.24E-06	3.54E-06

HSPB11	-1.27	1.07E-14	6.41E-14
LOC10013069	-1.27	4.90E-05	0.00011568
SSR4	-1.27	7.98E-27	1.02E-25
CDH1	-1.27	8.86E-78	6.40E-76
BTC	-1.27	3.52E-06	9.61E-06
LTBP4	-1.27	1.37E-46	4.31E-45
PAFAH1B2	-1.27	4.24E-29	6.17E-28
CDC42BPG	-1.27	4.73E-12	2.27E-11
PRIM1	-1.26	4.05E-29	5.91E-28
ATP2A3	-1.26	2.77E-12	1.36E-11
CDH3	-1.26	5.89E-81	4.62E-79
CLDN7	-1.26	5.56E-24	6.10E-23
BCL7C	-1.26	3.29E-25	3.90E-24
PSMA5	-1.26	3.93E-52	1.51E-50
RAD51B	-1.26	4.29E-10	1.74E-09
COL4A3	-1.26	0.00014076	0.00031361
SMARCD3	-1.26	1.00E-07	3.23E-07
BIN1	-1.26	1.20E-17	9.02E-17
KIFAP3	-1.26	1.18E-22	1.19E-21
AQP3	-1.26	4.77E-07	1.44E-06
SLC30A5	-1.26	1.03E-11	4.80E-11
NUCB1	-1.25	4.33E-70	2.66E-68
SCP2	-1.25	3.75E-11	1.67E-10
EML3	-1.25	1.98E-38	4.45E-37
LOC283038	-1.25	9.39E-07	2.73E-06
F13A1	-1.25	1.36E-35	2.68E-34
EMID1	-1.25	5.44E-08	1.79E-07
ARHGEF25	-1.25	2.70E-11	1.22E-10
JUNB	-1.25	7.49E-64	3.79E-62
CCNG2	-1.25	1.65E-20	1.47E-19
RPS6KB2	-1.25	7.55E-23	7.76E-22
MRPL39	-1.24	2.68E-29	3.94E-28
PAX6	-1.24	7.85E-06	2.05E-05
ILVBL	-1.24	3.01E-45	8.98E-44
MOSPD3	-1.24	6.98E-17	4.97E-16
TMEM63B	-1.24	6.17E-57	2.66E-55
CAST	-1.24	6.46E-106	8.17E-104
MFSD3	-1.24	1.13E-14	6.74E-14
GALT	-1.24	4.25E-17	3.05E-16
CKLF	-1.24	3.92E-28	5.45E-27
CREG2	-1.24	0.00011654	0.00026274
SLC27A1	-1.24	1.68E-11	7.71E-11

ERGIC3	-1.24	6.85E-77	4.86E-75
MAGED2	-1.24	6.70E-35	1.28E-33
CTAGE5	-1.23	1.93E-27	2.58E-26
KLC4	-1.23	1.83E-07	5.73E-07
BCAM	-1.23	3.50E-42	9.14E-41
ABCA5	-1.23	3.86E-07	1.17E-06
TMEM161A	-1.23	1.18E-16	8.25E-16
ACSM3	-1.23	6.48E-08	2.12E-07
LCMT1	-1.23	7.63E-14	4.28E-13
CHIC2	-1.23	2.82E-06	7.80E-06
CFD	-1.23	2.14E-06	6.01E-06
IRF6	-1.23	2.80E-11	1.26E-10
RPLP1	-1.23	1.61E-164	4.73E-162
ARHGEF26-AS	-1.23	1.40E-06	3.99E-06
KDELC1	-1.22	1.11E-09	4.32E-09
LOC1001310[illegible]	-1.22	4.85E-07	1.46E-06
TMC6	-1.22	1.25E-15	8.06E-15
FMO5	-1.22	0.00115251	0.0022464
TMEM141	-1.22	2.60E-28	3.64E-27
LIG1	-1.22	8.40E-25	9.65E-24
LOC1005081[illegible]	-1.22	0.00037752	0.00079158
GCAT	-1.22	5.35E-07	1.60E-06
CTSH	-1.22	5.86E-25	6.82E-24
PDZD4	-1.22	8.69E-05	0.00019878
OGDHL	-1.22	2.46E-05	6.01E-05
BHLHE40	-1.21	5.70E-90	5.50E-88
GAA	-1.21	2.66E-28	3.72E-27
PTK2B	-1.21	3.38E-12	1.65E-11
SERPINI1	-1.21	1.38E-08	4.83E-08
LOC1005069[illegible]	-1.21	1.34E-07	4.28E-07
TCN2	-1.21	1.29E-12	6.52E-12
BCAS3	-1.21	2.03E-20	1.81E-19
FBLN1	-1.21	2.30E-12	1.13E-11
BMF	-1.21	4.76E-08	1.58E-07
TRMT11	-1.20	1.67E-13	9.07E-13
IMPDH2	-1.20	4.96E-86	4.32E-84
ASL	-1.20	4.40E-31	6.93E-30
RPS14	-1.20	9.44E-145	2.30E-142
EML2	-1.20	2.98E-13	1.58E-12
LRTOMT	-1.20	3.70E-05	8.86E-05
LOC144481	-1.20	0.0002965	0.00063253
ODF2L	-1.20	8.65E-24	9.37E-23

SORBS1	-1.20	3.33E-10	1.36E-09
MAN2B1	-1.19	2.17E-40	5.33E-39
RABAC1	-1.19	2.30E-32	3.88E-31
SLC44A3	-1.19	3.88E-07	1.18E-06
ZNF385C	-1.19	1.05E-05	2.71E-05
HCFC1R1	-1.19	1.10E-50	4.02E-49
ITGB3BP	-1.19	5.55E-12	2.66E-11
FKBP10	-1.19	1.76E-17	1.30E-16
MMP11	-1.19	7.32E-06	1.92E-05
NSMCE1	-1.19	4.42E-24	4.88E-23
C17orf49	-1.19	1.34E-35	2.64E-34
RPL24	-1.19	6.99E-76	4.88E-74
SLC43A1	-1.19	2.80E-05	6.81E-05
PPP1R7	-1.19	6.06E-30	9.16E-29
ZNF467	-1.19	2.10E-10	8.73E-10
COX5B	-1.18	1.71E-36	3.47E-35
ERI2	-1.18	6.85E-07	2.02E-06
TMEM67	-1.18	1.95E-13	1.05E-12
ARHGEF16	-1.18	2.96E-11	1.33E-10
PIBF1	-1.18	4.61E-13	2.42E-12
PLD3	-1.18	8.54E-33	1.47E-31
MPND	-1.18	2.41E-06	6.68E-06
TNFAIP2	-1.18	4.37E-130	8.24E-128
ERCC2	-1.18	5.85E-29	8.44E-28
IQSEC2	-1.18	3.77E-07	1.15E-06
IFITM3	-1.18	9.27E-19	7.46E-18
GATS	-1.18	3.38E-08	1.14E-07
IRF7	-1.18	1.30E-07	4.14E-07
PFDN5	-1.17	6.91E-75	4.70E-73
SLC37A1	-1.17	5.99E-06	1.59E-05
STAG3	-1.17	0.00143677	0.00276128
MTHFD2L	-1.17	4.06E-05	9.68E-05
PIM3	-1.17	4.90E-47	1.57E-45
JAK3	-1.17	1.88E-10	7.85E-10
NQO2	-1.17	1.88E-26	2.36E-25
MPZL2	-1.17	2.63E-27	3.48E-26
RYR1	-1.17	0.00028966	0.00061907
KLHDC2	-1.17	3.54E-70	2.20E-68
ECH1	-1.17	2.22E-64	1.14E-62
TTC35	-1.17	2.75E-22	2.70E-21
ADAMTSL4	-1.17	3.16E-24	3.53E-23
GDPD5	-1.17	2.73E-10	1.12E-09

KLC3	-1.17	2.73E-07	8.43E-07
LRRC23	-1.16	3.12E-07	9.55E-07
ZP3	-1.16	6.34E-10	2.52E-09
SSBP4	-1.16	4.62E-08	1.53E-07
RAB11FIP4	-1.16	1.21E-12	6.13E-12
EGFL7	-1.16	5.96E-26	7.31E-25
AAAS	-1.16	8.12E-35	1.55E-33
PNKP	-1.16	3.33E-13	1.76E-12
ZNF695	-1.16	2.02E-07	6.32E-07
CTU2	-1.16	2.85E-11	1.28E-10
DDX43	-1.16	6.38E-18	4.87E-17
ALDH7A1	-1.16	1.57E-50	5.69E-49
GSTM4	-1.16	2.07E-13	1.11E-12
LIMK2	-1.16	2.52E-10	1.05E-09
DICER1-AS1	-1.15	0.00096542	0.00190612
C9orf46	-1.15	8.69E-12	4.08E-11
MST1P9	-1.15	0.0009873	0.0019478
C22orf26	-1.15	0.00010364	0.00023478
C1QL1	-1.15	3.15E-14	1.83E-13
SERINC5	-1.15	1.28E-05	3.25E-05
CGN	-1.15	7.58E-44	2.13E-42
SCN9A	-1.15	7.91E-06	2.06E-05
ASPSCR1	-1.15	1.96E-15	1.24E-14
FRY	-1.15	0.0022883	0.00425677
MACROD2	-1.15	0.00198662	0.00373458
HERC5	-1.15	1.21E-18	9.65E-18
MAP4K2	-1.15	3.93E-18	3.06E-17
SDR16C5	-1.15	1.36E-14	8.10E-14
RPS16	-1.15	5.36E-127	9.54E-125
SLC27A2	-1.15	6.79E-16	4.49E-15
MIPEP	-1.15	3.40E-11	1.51E-10
EFCAB11	-1.15	6.77E-05	0.00015703
U2AF1L4	-1.15	1.38E-07	4.37E-07
CHPT1	-1.14	4.60E-47	1.47E-45
EVI5L	-1.14	1.27E-18	1.02E-17
MED30	-1.14	5.92E-12	2.82E-11
LOC1001328[illegible]	-1.14	8.66E-05	0.00019828
DOCK11	-1.14	1.81E-12	9.02E-12
LOC728743	-1.14	5.04E-05	0.00011901
MMAB	-1.14	1.26E-27	1.70E-26
GPS2	-1.14	1.78E-32	3.01E-31
C8orf59	-1.14	5.78E-24	6.33E-23

VAV1	-1.14	5.18E-13	2.71E-12
CUEDC2	-1.14	2.37E-28	3.34E-27
GSN	-1.14	5.24E-32	8.68E-31
C4orf34	-1.14	2.31E-27	3.07E-26
RASA2	-1.14	1.63E-12	8.17E-12
LTA4H	-1.14	1.59E-69	9.46E-68
IFT88	-1.14	2.72E-06	7.53E-06
NFKBID	-1.14	0.00079455	0.00159213
KRT86	-1.14	0.00084814	0.00169066
LRRC45	-1.13	3.13E-15	1.95E-14
TP53TG1	-1.13	1.81E-05	4.50E-05
BRE	-1.13	1.29E-39	3.09E-38
AIG1	-1.13	1.40E-15	8.99E-15
SCARA5	-1.13	0.00143361	0.00275606
ING4	-1.13	6.52E-08	2.13E-07
SH3YL1	-1.13	5.21E-19	4.28E-18
CHAC1	-1.13	1.51E-16	1.05E-15
PHKB	-1.13	5.99E-58	2.64E-56
RPH3AL	-1.13	3.28E-18	2.56E-17
GARS	-1.13	8.59E-98	9.37E-96
MAD2L2	-1.13	7.50E-23	7.72E-22
TMEM173	-1.13	2.44E-28	3.44E-27
UQCRC1	-1.13	1.12E-74	7.53E-73
CCDC53	-1.13	2.39E-18	1.88E-17
NUP107	-1.13	3.10E-45	9.22E-44
EMR2	-1.12	0.00052312	0.00107585
OCIAD2	-1.12	8.90E-30	1.34E-28
C6orf203	-1.12	7.28E-06	1.91E-05
UBE2L6	-1.12	7.95E-32	1.30E-30
IK	-1.12	5.48E-46	1.69E-44
LIMCH1	-1.12	1.21E-62	5.91E-61
HIBCH	-1.12	2.94E-09	1.10E-08
MAP2	-1.12	0.00056187	0.00115012
CSTF3	-1.12	7.83E-10	3.10E-09
TPM1	-1.12	2.00E-56	8.53E-55
PTMS	-1.12	8.91E-72	5.69E-70
LPPR3	-1.12	1.04E-09	4.06E-09
MFSD4	-1.12	0.00035403	0.00074606
FDXR	-1.12	6.11E-11	2.67E-10
AS3MT	-1.12	0.00022412	0.00048574
UGGT2	-1.11	5.22E-12	2.51E-11
GSTK1	-1.11	7.32E-39	1.69E-37

RGL1	-1.11	0.00083612	0.0016675
PELI2	-1.11	1.15E-05	2.93E-05
PAPPA2	-1.11	0.00034587	0.00072959
MSI2	-1.11	8.61E-12	4.04E-11
PPA2	-1.11	1.20E-33	2.18E-32
NEBL	-1.11	6.25E-10	2.49E-09
SURF1	-1.11	2.56E-28	3.60E-27
OPLAH	-1.11	1.85E-17	1.36E-16
THBS3	-1.10	3.25E-17	2.35E-16
NNMT	-1.10	1.44E-89	1.37E-87
PLEKHH3	-1.10	4.06E-30	6.16E-29
ISG20	-1.10	3.86E-11	1.71E-10
SCNN1A	-1.10	2.75E-27	3.63E-26
EIF3M	-1.10	5.67E-49	1.97E-47
GKAP1	-1.10	3.64E-06	9.94E-06
IFI30	-1.10	6.70E-05	0.00015565
GSDMD	-1.10	1.08E-26	1.37E-25
AARS	-1.10	4.73E-144	1.11E-141
TNIP1	-1.10	1.24E-70	7.76E-69
AP4M1	-1.10	2.94E-06	8.10E-06
MSLN	-1.10	1.19E-12	6.06E-12
ALPK1	-1.10	1.50E-21	1.42E-20
CTH	-1.10	6.92E-07	2.04E-06
MED25	-1.10	5.07E-19	4.17E-18
VWDE	-1.10	2.67E-12	1.31E-11
HPS1	-1.10	6.85E-14	3.87E-13
RPL5	-1.10	4.78E-83	3.85E-81
EIF2D	-1.10	6.40E-39	1.49E-37
TMEM92	-1.10	1.38E-05	3.48E-05
SIL1	-1.09	2.06E-19	1.73E-18
ADAMTSL3	-1.09	6.15E-05	0.00014357
LOC1006527(	-1.09	0.00407571	0.00726845
VWA1	-1.09	1.73E-15	1.10E-14
WDR78	-1.09	0.00428399	0.0076045
CRELD1	-1.09	4.24E-09	1.55E-08
GNB1L	-1.09	0.00017728	0.0003895
SIPA1	-1.09	2.20E-23	2.32E-22
DCTN3	-1.09	1.29E-24	1.47E-23
CD9	-1.09	1.60E-80	1.25E-78
ZDHHC1	-1.09	5.22E-05	0.00012288
SCNM1	-1.09	2.35E-26	2.94E-25
PTTG1	-1.09	4.67E-52	1.79E-50

CCDC85B	-1.09	1.08E-64	5.64E-63
ATP5H	-1.09	4.41E-44	1.25E-42
C6orf72	-1.09	4.24E-23	4.42E-22
MYZAP	-1.09	4.45E-05	0.00010574
NDUFB8	-1.09	1.36E-40	3.40E-39
C17orf57	-1.09	4.51E-05	0.00010691
BMP1	-1.08	8.74E-25	1.00E-23
CCDC106	-1.08	2.77E-15	1.74E-14
WDR18	-1.08	1.66E-48	5.64E-47
RAB4B	-1.08	1.75E-12	8.74E-12
OCEL1	-1.08	2.62E-10	1.08E-09
MR1	-1.08	1.12E-14	6.69E-14
LONP1	-1.08	3.16E-84	2.60E-82
CTSD	-1.08	9.17E-43	2.46E-41
GAMT	-1.08	6.82E-10	2.71E-09
FIBP	-1.08	4.14E-37	8.72E-36
DRAP1	-1.08	8.28E-61	3.89E-59
YPEL2	-1.08	1.11E-08	3.92E-08
WBSCR27	-1.08	6.44E-05	0.00014993
DNAJC4	-1.08	1.75E-16	1.21E-15
SFI1	-1.07	5.22E-09	1.90E-08
MARS	-1.07	8.02E-69	4.70E-67
TMEM107	-1.07	4.45E-07	1.34E-06
MITD1	-1.07	6.32E-14	3.58E-13
DPY19L1P1	-1.07	2.96E-05	7.16E-05
COPE	-1.07	6.85E-26	8.37E-25
MLXIPL	-1.07	2.12E-06	5.94E-06
LOXL2	-1.07	1.95E-99	2.17E-97
ATG16L2	-1.07	9.79E-06	2.52E-05
CALB2	-1.07	9.17E-06	2.37E-05
ZNF677	-1.07	0.00034936	0.00073647
METTL5	-1.07	5.81E-30	8.79E-29
ERP44	-1.07	1.75E-36	3.56E-35
SLMO2-ATP5I	-1.07	1.92E-75	1.33E-73
MIA-RAB4B	-1.07	4.40E-12	2.12E-11
NOSIP	-1.07	1.39E-16	9.67E-16
TBC1D22A	-1.07	1.85E-22	1.84E-21
CHMP5	-1.07	1.79E-57	7.76E-56
LOC1005057[illegible]	-1.06	5.56E-05	0.00013038
NPRL2	-1.06	3.43E-10	1.40E-09
PREX1	-1.06	4.99E-06	1.34E-05
SLC39A11	-1.06	6.36E-20	5.51E-19

PNPLA2	-1.06	1.26E-29	1.87E-28
CALCOCO1	-1.06	1.07E-23	1.16E-22
CALHM2	-1.06	6.43E-14	3.64E-13
RNF130	-1.06	1.03E-35	2.03E-34
TSTD1	-1.06	1.23E-13	6.77E-13
PKN1	-1.06	1.97E-62	9.57E-61
B4GALNT4	-1.06	2.64E-06	7.32E-06
MAD1L1	-1.06	2.98E-15	1.86E-14
PCIF1	-1.06	2.88E-27	3.80E-26
IQCH	-1.06	9.52E-05	0.00021674
CARS	-1.06	7.50E-45	2.18E-43
SRPX	-1.06	4.83E-08	1.60E-07
HIST1H1C	-1.06	3.95E-43	1.07E-41
MTHFD1L	-1.06	5.42E-22	5.23E-21
ZNF277	-1.06	5.64E-18	4.33E-17
KIAA1704	-1.06	5.83E-09	2.11E-08
COQ6	-1.06	2.78E-16	1.89E-15
HEXDC	-1.06	3.04E-07	9.32E-07
C16orf13	-1.06	1.10E-23	1.18E-22
ARHGEF1	-1.05	1.94E-54	7.83E-53
LOC1001292	-1.05	0.00048227	0.00099768
RPL3	-1.05	1.20E-156	3.22E-154
KPNA5	-1.05	6.75E-09	2.43E-08
POLD1	-1.05	3.25E-37	6.91E-36
MXD4	-1.05	1.52E-25	1.82E-24
ABLIM3	-1.05	1.05E-19	8.97E-19
TECPR1	-1.05	1.04E-11	4.87E-11
SEZ6L2	-1.05	1.00E-15	6.52E-15
CKLF-CMTM1	-1.05	1.52E-12	7.63E-12
FKBP2	-1.05	1.63E-18	1.29E-17
INPP5B	-1.05	1.77E-27	2.36E-26
ME3	-1.05	1.78E-15	1.13E-14
RPS13	-1.05	3.30E-34	6.17E-33
PARP9	-1.05	1.26E-12	6.41E-12
SLC37A2	-1.05	0.00327604	0.00592933
DOCK6	-1.05	3.32E-21	3.07E-20
LOC1005063	-1.04	2.13E-06	5.98E-06
RPS27	-1.04	5.31E-142	1.20E-139
AGFG2	-1.04	1.14E-07	3.65E-07
CSF1	-1.04	8.05E-52	3.04E-50
EGF	-1.04	0.00021172	0.0004602
APOL6	-1.04	5.21E-21	4.78E-20

MAGOH	-1.04	6.60E-13	3.43E-12
ADCK5	-1.04	0.00030288	0.00064473
EXOSC8	-1.04	5.97E-15	3.64E-14
SLC5A10	-1.04	2.93E-05	7.09E-05
MVD	-1.04	3.28E-37	6.96E-36
ANKRA2	-1.04	5.48E-10	2.20E-09
FCHSD1	-1.04	1.37E-08	4.78E-08
ISG15	-1.04	7.82E-15	4.73E-14
RNF123	-1.04	1.12E-16	7.87E-16
MTMR11	-1.04	3.97E-22	3.87E-21
IMPA2	-1.04	2.62E-37	5.61E-36
KYNU	-1.04	1.00E-15	6.52E-15
RMND5B	-1.03	7.99E-17	5.66E-16
FAM171A2	-1.03	1.17E-05	3.00E-05
VPS28	-1.03	1.05E-16	7.35E-16
CDKN1C	-1.03	0.00102621	0.00201826
NR1H3	-1.03	0.00063714	0.00129518
RPN2	-1.03	5.10E-113	7.58E-111
SERGEF	-1.03	8.22E-08	2.67E-07
IFT172	-1.03	2.27E-06	6.35E-06
PLA2R1	-1.03	5.14E-06	1.38E-05
COPG2	-1.03	7.54E-24	8.20E-23
RQCD1	-1.03	4.26E-15	2.63E-14
CATSPER1	-1.03	0.00857707	0.01452954
CTSF	-1.03	1.68E-18	1.33E-17
MRPL47	-1.03	1.71E-18	1.35E-17
TMEM41B	-1.02	1.60E-07	5.06E-07
NDUFS4	-1.02	8.74E-15	5.26E-14
FOXO4	-1.02	4.35E-08	1.45E-07
CNKSR1	-1.02	1.84E-10	7.71E-10
GSTM3	-1.02	1.28E-17	9.55E-17
LOC1001339[illegible]	-1.02	0.00250842	0.00463487
AGRN	-1.02	5.25E-86	4.55E-84
UGCG	-1.02	5.07E-50	1.81E-48
TMEM37	-1.02	0.00057895	0.00118165
ABCA2	-1.02	1.57E-40	3.89E-39
TMEM14A	-1.02	2.36E-31	3.78E-30
CTTN	-1.02	4.06E-70	2.50E-68
ADK	-1.02	6.84E-72	4.41E-70
PALM	-1.02	1.98E-10	8.25E-10
DLEU2L	-1.02	0.00128818	0.00249583
CIR1	-1.02	3.10E-11	1.39E-10

LMX1B	-1.02	0.00128165	0.00248432
TKT	-1.02	1.02E-74	6.90E-73
RPGR	-1.02	9.61E-06	2.48E-05
PRKCG	-1.02	0.00323903	0.00586738
PRDX5	-1.02	5.65E-69	3.32E-67
FCGRT	-1.01	1.51E-18	1.21E-17
IFT74	-1.01	2.83E-09	1.06E-08
RAB17	-1.01	1.78E-07	5.61E-07
KLHL24	-1.01	2.36E-09	8.91E-09
C3orf26	-1.01	3.82E-15	2.36E-14
LINC00467	-1.01	6.34E-11	2.76E-10
TMEM44	-1.01	3.93E-09	1.45E-08
MDP1	-1.01	0.00037756	0.00079158
ANXA11	-1.01	8.78E-66	4.77E-64
LGSN	-1.01	3.60E-09	1.33E-08
CMC1	-1.01	1.74E-09	6.68E-09
DAK	-1.01	6.60E-38	1.45E-36
ABCA13	-1.01	1.06E-08	3.73E-08
RIMS4	-1.01	2.04E-24	2.31E-23
LOC1005075(	-1.01	0.00035855	0.00075508
IFIH1	-1.01	1.96E-08	6.75E-08
BBS9	-1.01	2.77E-09	1.04E-08
RBX1	-1.00	6.42E-18	4.90E-17
ASB9	-1.00	3.59E-05	8.62E-05
TRIOBP	-1.00	1.00E-34	1.90E-33
ARMC9	-1.00	0.00010529	0.00023832
LOC1005054(	-1.00	0.00802334	0.01367211
C1orf192	-1.00	1.34E-06	3.83E-06
PHYHD1	-1.00	5.22E-09	1.90E-08
VPS33B	-1.00	2.13E-09	8.08E-09
FAM98C	-1.00	0.00075302	0.00151418
PTPRE	-1.00	2.10E-37	4.50E-36
SPTB	1.00	2.81E-18	2.20E-17
GAB2	1.00	2.09E-10	8.69E-10
VEGFC	1.00	9.01E-25	1.03E-23
GPR137C	1.01	3.36E-10	1.37E-09
TMEM154	1.01	0.00202291	0.00379829
GOLGA6L5	1.01	1.82E-05	4.51E-05
GJA1	1.01	8.09E-15	4.89E-14
UBAC2-AS1	1.02	4.54E-06	1.22E-05
CRMP1	1.02	6.78E-05	0.00015726
HCN2	1.02	0.0008798	0.00174772

RNF169	1.02	6.52E-52	2.48E-50
ICMT	1.02	7.88E-119	1.28E-116
MXD1	1.02	5.06E-18	3.90E-17
TARDBP	1.02	6.53E-95	6.82E-93
SPIN3	1.03	5.37E-14	3.07E-13
ETNK1	1.03	8.47E-08	2.75E-07
RPS26	1.03	2.91E-86	2.57E-84
ANKRD23	1.03	3.33E-06	9.12E-06
WNT5B	1.03	2.19E-07	6.83E-07
C17orf79	1.03	1.04E-30	1.62E-29
SRRM2	1.03	2.69E-130	5.15E-128
HOXA9	1.03	8.77E-05	0.00020045
FLNC	1.03	6.85E-129	1.27E-126
ARID3B	1.03	1.83E-24	2.07E-23
FAM72D	1.03	1.89E-43	5.18E-42
LOC1001267[illegible]	1.03	0.0003268	0.00069226
FOXO3	1.04	2.61E-27	3.46E-26
MMP14	1.04	3.29E-112	4.84E-110
HNRNPU-AS1	1.04	4.48E-21	4.12E-20
EIF5A	1.04	1.51E-116	2.33E-114
RFTN1	1.04	1.19E-15	7.68E-15
DDX12P	1.04	6.00E-07	1.78E-06
NEU3	1.04	4.44E-18	3.44E-17
ZNF850	1.04	5.63E-14	3.21E-13
ANKRD1	1.04	1.39E-36	2.84E-35
PVRL1	1.04	2.74E-96	2.94E-94
AHNAK	1.04	3.21E-46	9.92E-45
C1orf216	1.04	1.96E-25	2.34E-24
EIF5A2	1.05	1.38E-11	6.35E-11
OXTR	1.05	6.18E-14	3.51E-13
NIPA1	1.05	3.23E-76	2.28E-74
DPY19L2	1.05	4.40E-06	1.19E-05
BMPER	1.05	6.86E-17	4.89E-16
KLHL11	1.05	8.96E-11	3.84E-10
POU4F1	1.05	0.00486679	0.00856685
C1orf63	1.05	9.12E-20	7.83E-19
ANKRD13A	1.05	2.37E-132	4.83E-130
ZNF514	1.06	2.50E-17	1.83E-16
41337	1.06	3.77E-42	9.80E-41
LCAT	1.06	1.35E-07	4.30E-07
TMEM156	1.06	1.02E-22	1.04E-21
BDNF	1.06	1.21E-43	3.36E-42

GFOD1	1.06	4.72E-05	0.00011178
AOC2	1.06	1.27E-07	4.06E-07
MOB3B	1.06	4.81E-11	2.12E-10
ANKRD50	1.06	9.52E-51	3.49E-49
TSPYL4	1.06	2.12E-32	3.60E-31
CCDC144A	1.07	0.00135783	0.00261873
LINC00338	1.07	5.34E-14	3.05E-13
EXOG	1.07	3.90E-16	2.62E-15
LOC254559	1.07	0.00221121	0.00412368
ZNF600	1.07	1.99E-15	1.26E-14
NSUN5P1	1.07	7.00E-06	1.84E-05
ZNF407	1.07	3.84E-16	2.58E-15
41520	1.07	3.29E-17	2.38E-16
KIAA1462	1.07	2.27E-59	1.03E-57
DNASE1	1.08	8.43E-07	2.46E-06
ZNF121	1.08	7.33E-21	6.67E-20
GDAP1	1.08	2.73E-48	9.14E-47
LOC158257	1.08	0.00113866	0.00222145
LOC1001283[illegible]	1.08	6.02E-14	3.42E-13
C17orf51	1.08	8.28E-42	2.13E-40
ZNF841	1.08	2.64E-14	1.54E-13
LYPD5	1.08	0.00329378	0.00595972
CDC42EP2	1.08	3.24E-31	5.15E-30
METTL12	1.09	7.14E-09	2.56E-08
PKP1	1.09	1.23E-14	7.33E-14
C4orf19	1.09	2.56E-09	9.64E-09
PEA15	1.09	1.13E-108	1.51E-106
CLSPN	1.09	1.54E-49	5.43E-48
CHORDC1	1.09	5.98E-43	1.61E-41
EFNB2	1.09	3.86E-17	2.78E-16
PEG10	1.10	3.31E-110	4.61E-108
SLC6A17	1.10	7.42E-10	2.94E-09
PPP1R3E	1.10	1.58E-14	9.37E-14
LTB4R	1.10	2.25E-10	9.35E-10
FICD	1.10	8.24E-08	2.68E-07
MBOAT2	1.10	4.28E-83	3.47E-81
KLHL21	1.11	6.50E-42	1.68E-40
ZNF799	1.11	6.73E-10	2.68E-09
FOXL1	1.11	8.31E-13	4.28E-12
ZNF267	1.11	1.04E-26	1.32E-25
FCRLB	1.11	0.00024665	0.00053177
CDK5R1	1.11	2.34E-11	1.06E-10

CELF4	1.11	5.18E-06	1.39E-05
FAM35A	1.11	6.76E-47	2.14E-45
CSTB	1.11	5.01E-126	8.79E-124
FAM101B	1.11	1.14E-107	1.50E-105
LOC1005056[illegible]	1.12	0.00018883	0.00041294
CORO2B	1.12	2.21E-11	1.00E-10
MGC12982	1.12	0.00022881	0.00049505
XRCC2	1.12	1.00E-37	2.18E-36
SLC5A3	1.12	2.54E-55	1.06E-53
LOC646719	1.12	5.01E-39	1.17E-37
LOC1002890[illegible]	1.13	1.41E-06	4.01E-06
MYB	1.13	4.98E-05	0.00011758
ZNF589	1.13	1.18E-18	9.46E-18
PLEKHM1	1.13	1.21E-06	3.47E-06
TMED10P1	1.13	5.73E-13	2.99E-12
PPAPDC1A	1.13	4.21E-20	3.69E-19
SHISA7	1.13	5.13E-05	0.00012081
G0S2	1.14	2.33E-41	5.93E-40
ZNF587	1.14	1.48E-31	2.40E-30
ADAM1	1.14	5.79E-08	1.90E-07
LOC1005066[illegible]	1.14	5.31E-49	1.85E-47
LOC1005070[illegible]	1.14	3.72E-16	2.50E-15
MALAT1	1.15	4.48E-105	5.45E-103
LOC147804	1.15	2.66E-17	1.94E-16
LOC1001290[illegible]	1.15	1.32E-08	4.62E-08
GEM	1.15	1.39E-12	7.02E-12
RPSAP9	1.15	1.89E-06	5.33E-06
LOC728643	1.15	6.05E-07	1.80E-06
ZFP112	1.15	2.33E-11	1.05E-10
MSTO2P	1.15	1.14E-09	4.42E-09
LINC00115	1.15	0.00035866	0.00075518
ENC1	1.15	1.67E-60	7.80E-59
TMPPE	1.16	6.99E-05	0.00016195
FREM2	1.16	3.12E-05	7.54E-05
MGC21881	1.16	2.03E-27	2.71E-26
PLEKHA8P1	1.16	7.93E-07	2.33E-06
HIST1H2BJ	1.16	6.53E-06	1.72E-05
TLR2	1.17	1.91E-05	4.74E-05
ZEB1	1.17	8.36E-35	1.59E-33
ZNF185	1.17	1.19E-88	1.10E-86
FLG	1.17	2.12E-16	1.46E-15
THSD1	1.17	1.41E-10	5.93E-10

SLC35G2	1.17	1.43E-38	3.23E-37
TTN	1.18	1.39E-14	8.28E-14
SMTN	1.18	3.58E-48	1.19E-46
FOXO3B	1.18	8.48E-11	3.64E-10
CREB5	1.18	7.47E-17	5.32E-16
CHRNA3	1.18	0.00086618	0.00172391
ABHD16B	1.18	1.82E-16	1.26E-15
IL11	1.18	2.13E-27	2.83E-26
LPAR1	1.18	7.67E-85	6.42E-83
PRDM13	1.19	4.92E-08	1.63E-07
LOC654342	1.19	8.55E-07	2.50E-06
SLC4A4	1.19	7.87E-52	2.99E-50
SPRN	1.19	1.62E-12	8.16E-12
FAM86DP	1.19	5.67E-15	3.46E-14
JPH1	1.20	6.44E-24	7.05E-23
KLF12	1.20	3.24E-19	2.70E-18
FRMD6	1.20	4.54E-134	9.57E-132
CLDN15	1.20	2.03E-15	1.29E-14
ZNF594	1.20	9.04E-16	5.90E-15
LOC344595	1.20	3.66E-09	1.35E-08
MAMLD1	1.21	1.85E-22	1.84E-21
SOX9	1.21	1.51E-36	3.08E-35
NBPF9	1.21	3.25E-33	5.74E-32
PNN	1.21	2.15E-137	4.68E-135
LOC401588	1.21	1.58E-09	6.09E-09
LOC642846	1.21	4.19E-08	1.40E-07
ZNF542	1.21	7.05E-33	1.22E-31
PPP1R11	1.21	6.15E-07	1.83E-06
ELOVL2	1.21	6.98E-91	6.94E-89
FOXC1	1.21	1.55E-05	3.89E-05
SRSF1	1.22	8.81E-211	3.38E-208
LOC646903	1.22	5.43E-05	0.00012761
KAL1	1.22	6.75E-19	5.50E-18
ENTPD7	1.22	3.51E-96	3.73E-94
ZNF767	1.22	1.22E-14	7.28E-14
DOK3	1.22	3.49E-06	9.52E-06
LOC645431	1.22	2.52E-05	6.15E-05
SON	1.22	4.15E-141	9.20E-139
YY2	1.22	8.44E-13	4.34E-12
LOX	1.23	1.57E-44	4.55E-43
ITGA2	1.23	3.40E-78	2.47E-76
FAM111B	1.23	1.47E-104	1.72E-102

GRPR	1.23	2.00E-13	1.08E-12
HOXB5	1.23	2.32E-21	2.17E-20
ZNF440	1.24	1.36E-16	9.45E-16
FAM86C1	1.24	2.97E-24	3.33E-23
XK	1.24	4.25E-25	4.98E-24
ALG10	1.24	5.81E-15	3.54E-14
RRS1	1.25	2.08E-107	2.66E-105
LOC1005071	1.25	2.06E-107	2.65E-105
DGCR11	1.25	4.61E-12	2.22E-11
C3orf52	1.25	5.96E-15	3.63E-14
RAB3B	1.25	4.37E-161	1.23E-158
ATAD3B	1.25	2.09E-20	1.86E-19
ZNF782	1.25	1.46E-09	5.64E-09
ZFHX2	1.25	3.73E-06	1.02E-05
CLCN4	1.25	8.51E-14	4.74E-13
LOC219731	1.26	1.37E-05	3.47E-05
LOC1001309	1.26	2.54E-19	2.13E-18
RP9P	1.26	8.20E-17	5.81E-16
NAP1L2	1.26	4.31E-09	1.58E-08
SACS	1.26	2.36E-115	3.60E-113
TUBB	1.26	1.87E-65	1.01E-63
LOC1005058	1.27	2.55E-07	7.89E-07
NPTX2	1.27	1.88E-05	4.66E-05
SPATA13	1.27	6.82E-25	7.89E-24
FAIM3	1.27	1.13E-29	1.69E-28
SEMA3A	1.28	8.24E-11	3.54E-10
RPL23AP7	1.29	1.12E-08	3.94E-08
RRP7B	1.29	1.47E-10	6.19E-10
HSPA8	1.30	1.75E-249	1.01E-246
FLJ37201	1.30	6.69E-05	0.00015539
ZNF326	1.30	4.54E-08	1.51E-07
STARD13	1.30	3.16E-13	1.67E-12
TJP2	1.30	1.97E-196	6.93E-194
BTBD6	1.30	4.12E-124	7.04E-122
WASH1	1.30	4.85E-12	2.33E-11
41334.1	1.31	6.93E-18	5.27E-17
GCET2	1.31	5.51E-06	1.47E-05
PTHLH	1.31	2.43E-09	9.16E-09
MAP6D1	1.32	5.75E-09	2.09E-08
FAM86B1	1.32	1.03E-09	4.04E-09
TNFRSF11A	1.32	1.88E-19	1.59E-18
LOC644656	1.32	2.58E-05	6.30E-05

LOC440300	1.33	4.86E-23	5.06E-22
RNF212	1.33	1.85E-48	6.27E-47
ACTC1	1.33	5.23E-06	1.40E-05
PTGER2	1.34	2.25E-13	1.21E-12
ZNF780A	1.34	6.95E-25	8.03E-24
ARHGAP24	1.34	1.19E-08	4.20E-08
ATF5	1.34	1.02E-38	2.33E-37
CCL5	1.34	2.90E-05	7.03E-05
DUSP7	1.34	2.62E-33	4.65E-32
AGPAT1	1.34	2.31E-09	8.73E-09
LOC149837	1.35	6.84E-24	7.48E-23
41334	1.37	0.00018962	0.00041459
TFRC	1.37	2.42E-212	9.54E-210
LOC440354	1.37	3.09E-08	1.04E-07
PIGW	1.37	5.29E-66	2.94E-64
USP32P1	1.37	6.08E-32	1.00E-30
PI4KAP1	1.38	8.35E-14	4.66E-13
C12orf34	1.38	6.13E-11	2.67E-10
MGC70870	1.39	1.64E-131	3.23E-129
SPIN2B	1.39	2.01E-08	6.91E-08
NEAT1	1.39	6.19E-158	1.70E-155
ARMCX4	1.40	1.03E-20	9.24E-20
NRIP3	1.40	1.38E-35	2.72E-34
HOXB6	1.40	3.43E-44	9.80E-43
PDIA3P	1.40	1.22E-16	8.49E-16
CHRM3	1.40	9.81E-11	4.19E-10
SPRY4	1.40	2.88E-13	1.53E-12
ZNF417	1.41	5.99E-15	3.64E-14
LOC154761	1.41	1.34E-06	3.82E-06
KBTBD8	1.41	8.09E-06	2.11E-05
LOC1002722	1.41	4.08E-06	1.11E-05
LOC646329	1.42	4.20E-06	1.14E-05
HIST1H4H	1.42	6.11E-10	2.44E-09
MORC2-AS1	1.43	6.03E-07	1.79E-06
FRMD6-AS1	1.43	1.60E-10	6.72E-10
RRN3P3	1.44	6.21E-11	2.71E-10
LOC1005074	1.45	2.52E-07	7.80E-07
GNRH1	1.45	6.87E-09	2.47E-08
PRG4	1.45	6.29E-19	5.15E-18
NBPF10	1.46	1.19E-16	8.33E-16
LOC401431	1.46	3.19E-14	1.85E-13
SPHK1	1.46	3.49E-24	3.90E-23

WASH3P	1.46	4.92E-15	3.01E-14
LOC595101	1.47	1.76E-08	6.07E-08
RPL23AP64	1.47	7.73E-06	2.02E-05
LOC730755	1.47	3.50E-08	1.17E-07
HOXB8	1.47	3.72E-29	5.43E-28
FERMT1	1.48	2.33E-119	3.83E-117
NEFM	1.48	7.19E-12	3.40E-11
DDIT4L	1.49	8.41E-27	1.08E-25
IL24	1.49	6.05E-25	7.01E-24
HTR7P1	1.50	2.48E-17	1.81E-16
MGC57346	1.51	6.78E-13	3.52E-12
ABL2	1.51	1.01E-203	3.76E-201
CD274	1.52	1.07E-62	5.28E-61
SMPD3	1.52	8.72E-11	3.74E-10
TMEM158	1.52	1.65E-22	1.65E-21
TUBA1C	1.52	7.55E-240	3.67E-237
MICA	1.52	9.63E-25	1.10E-23
ZNF114	1.52	1.21E-30	1.88E-29
AFG3L1P	1.52	2.22E-69	1.32E-67
TUBB1	1.53	9.84E-09	3.48E-08
B3GALT5	1.53	7.26E-06	1.91E-05
FGF5	1.53	1.10E-06	3.17E-06
SHISA9	1.54	2.02E-45	6.11E-44
EIF3CL	1.55	1.09E-21	1.04E-20
EIF3C	1.55	1.10E-21	1.05E-20
AGAP5	1.56	3.24E-07	9.91E-07
TUBA1B	1.56	9.76E-268	6.85E-265
GOLGA8B	1.58	4.47E-87	4.01E-85
MIR155HG	1.58	3.11E-08	1.05E-07
WASH5P	1.59	3.41E-55	1.41E-53
SGK196	1.59	2.44E-17	1.79E-16
ST20	1.59	1.11E-09	4.32E-09
LOC1001909[illegible]	1.59	7.06E-14	3.98E-13
OBFC2A	1.61	6.41E-137	1.37E-134
C11orf41	1.61	1.46E-12	7.35E-12
RNF182	1.61	2.55E-38	5.71E-37
BMS1P1	1.65	9.87E-07	2.86E-06
BMS1P5	1.65	9.95E-07	2.88E-06
DHRS4L2	1.65	5.82E-12	2.78E-11
PTGDR2	1.65	1.69E-16	1.17E-15
GPR89A	1.69	1.27E-18	1.02E-17
ICOSLG	1.69	1.74E-30	2.68E-29

ZNF443	1.70	4.32E-12	2.09E-11
DQX1	1.70	2.11E-10	8.78E-10
HERC2P2	1.72	3.42E-79	2.62E-77
GOLGA8A	1.73	5.35E-58	2.37E-56
C6orf141	1.76	9.64E-49	3.32E-47
LOC284454	1.76	7.89E-44	2.21E-42
LBH	1.77	1.24E-15	7.99E-15
TNFAIP3	1.79	1.88E-213	7.93E-211
NBPF16	1.80	3.34E-93	3.44E-91
HERC2P9	1.81	1.88E-18	1.49E-17
BCL2A1	1.82	3.93E-15	2.43E-14
CCZ1	1.82	1.92E-27	2.57E-26
IL1RL1	1.91	7.80E-19	6.31E-18
NAV3	1.93	1.62E-65	8.73E-64
AGAP6	1.96	3.65E-24	4.07E-23
KCNQ5	1.98	8.16E-32	1.33E-30
MMP1	2.01	4.87E-25	5.68E-24
LOC10028877	2.02	2.62E-14	1.53E-13
OPHN1	2.02	1.90E-68	1.10E-66
LOC10021600	2.06	1.01E-11	4.71E-11
HMGA2	2.12	4.43E-53	1.72E-51
LOC10013224	2.18	7.04E-16	4.65E-15
PFN1P2	2.24	3.85E-33	6.76E-32
LOC10050612	2.27	6.39E-19	5.23E-18
SRSF10	2.33	1.04E-22	1.05E-21
NBPF14	2.37	5.00E-81	3.96E-79
GKN2	2.61	4.33E-35	8.31E-34
FGB	2.87	1.46E-54	5.92E-53
ZCWPW2	4.12	6.58E-91	6.60E-89
