# Supplementary material for: Messenger RNA profile analysis deciphers new Esrrb responsive genes in prostate cancer cells
Source: BMC Mol Biol. 2015 Dec 1;16:21. doi: 10.1186/s12867-015-0049-1 (PMC4667504; doi:10.1186/s12867-015-0049-1)
Supplement: Supplementary file 2 — 10.1186/s12867-015-0049-1 Gene ontology analysis result. Table S2. Esrrb expression with DY131 treatment (control vs. Esrrb + DY131). [file 12867_2015_49_MOESM2_ESM.pdf]

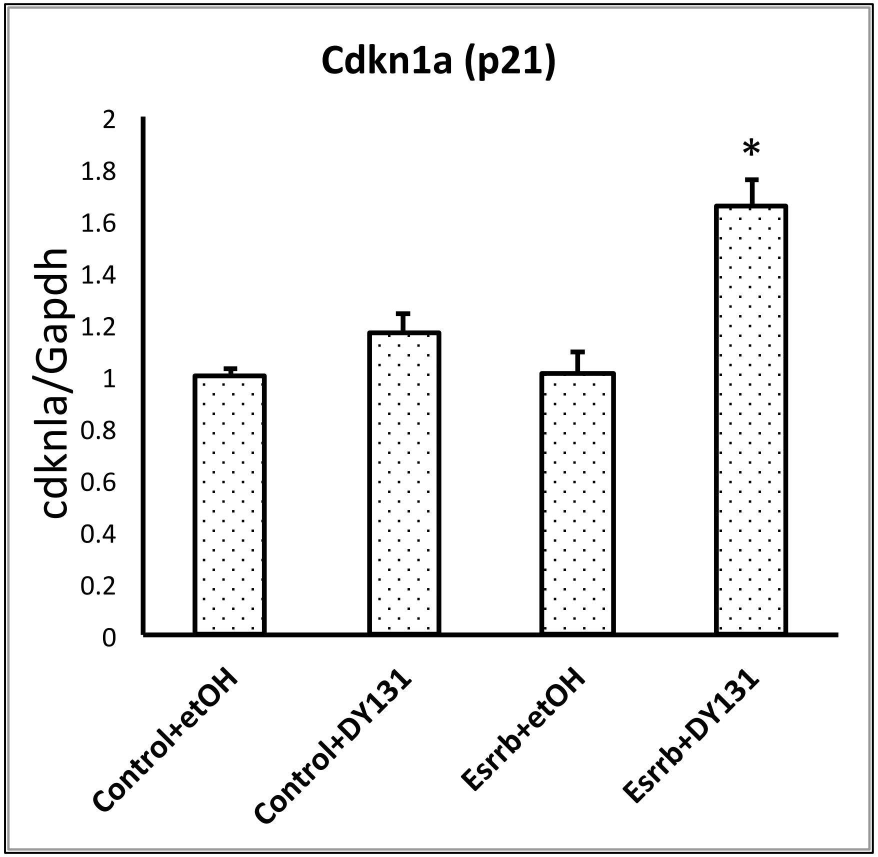
Cdkn1a (p21)
cdknla/Gapdh
2
1.8
1.6
1.4
1.2
1
0.8
0.6
0.4
0.2
0
*
Control+etOH
Control+DY131
Esrrb+etOH
Esrrb+DY131

Additional Figure 1. DY131-activated Esrrb regulates p21. qPCR analysis was performed to confirm p21 mRNA concentration regulation requires both Esrrb expression and DY131 treatment. * means $p<0.05$.

a

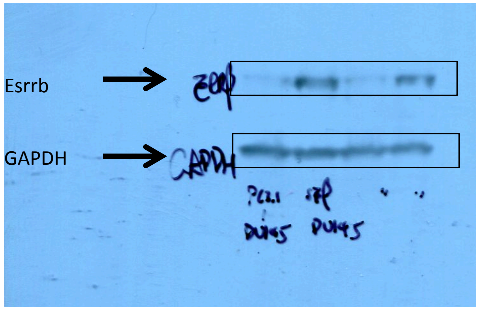

b

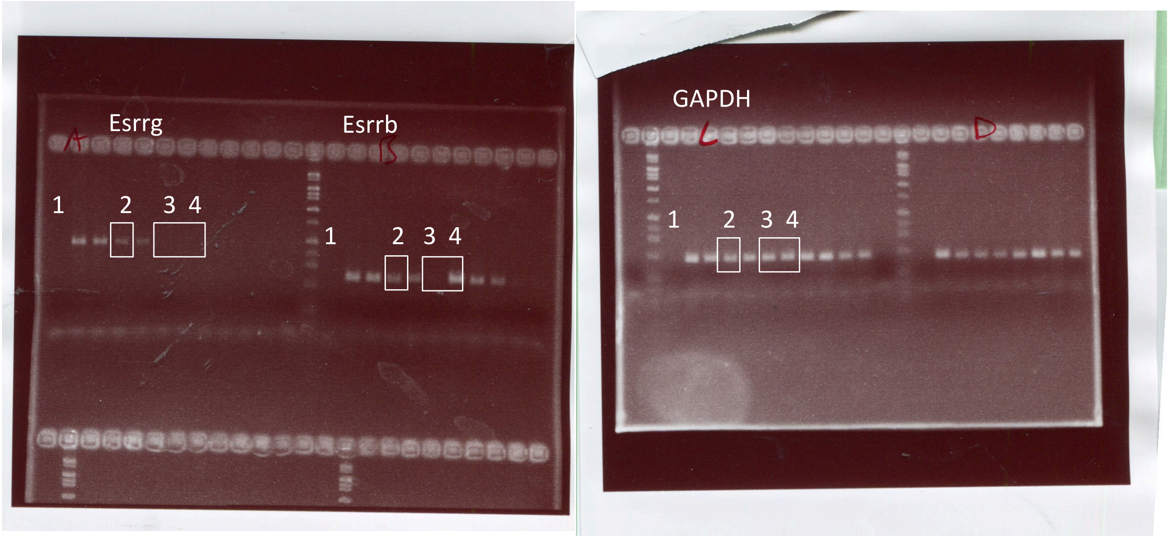

1. Water as reverse-transcription template
2. HEK293
3. DU145-pc3.1
4. DU145-Esrrb

Additional Figure 2. Full gel images. (a) Full blot image for Figure 1b. (b) Full gel image for Figure 1c.
